# Human Poisoning from Poisonous Higher Fungi: Focus on Analytical Toxicology and Case Reports in Forensic Toxicology

**DOI:** 10.3390/ph13120454

**Published:** 2020-12-11

**Authors:** Estelle Flament, Jérôme Guitton, Jean-Michel Gaulier, Yvan Gaillard

**Affiliations:** 1 Laboratory LAT LUMTOX, 07800 La Voulte sur Rhône, France; e.flament@latlumtox.com (E.F.); y.gaillard@latlumtox.com (Y.G.); 2 Laboratory of Pharmacology and Toxicology, Lyon-Sud University Hospital–Hospices Civil de Lyon, 69002 Pierre Bénite, France; 3 Department of Toxicology, Faculty of Pharmacy, University Claude Bernard, 69622 Lyon, France; 4 Department of Toxicology and Genopathy, Lille University Hospital, 59000 Lille, France; jeanmichel.gaulier@chru-lille.fr

**Keywords:** mushroom poisoning, mycotoxins, orellanine, analytical toxicology, amatoxins, forensic toxicology

## Abstract

Several families of higher fungi contain mycotoxins that cause serious or even fatal poisoning when consumed by humans. The aim of this review is to inventory, from an analytical point of view, poisoning cases linked with certain significantly toxic mycotoxins: orellanine, α- and β-amanitin, muscarine, ibotenic acid and muscimol, and gyromitrin. Clinicians are calling for the cases to be documented by toxicological analysis. This document is therefore a review of poisoning cases involving these mycotoxins reported in the literature and carries out an inventory of the analytical techniques available for their identification and quantification. It seems indeed that these poisonings are only rarely documented by toxicological analysis, due mainly to a lack of analytical methods in biological matrices. There are many reasons for this issue: the numerous varieties of mushroom involved, mycotoxins with different chemical structures, a lack of knowledge about distribution and metabolism. To sum up, we are faced with (i) obstacles to the documentation and interpretation of fatal (or non-fatal) poisoning cases and (ii) a real need for analytical methods of identifying and quantifying these mycotoxins (and their metabolites) in biological matrices.

## 1. Introduction

There is an extremely diverse range of fungi about which little is known. One million five hundred thousand species were known in 2002, 5.1 million in 2005, and the figure reached 13.5 million species in 2018. In reality, the exact number of fungal species on Earth is as yet unknown, since we are only aware of a tiny proportion of this diversity, of which only 100,000 species have been described [1]. Among these, there are about 5000 species of so-called higher fungi [2], those where the sporophore (the reproductive organ in fungi) is visible to the naked eye. Of these, a few dozen species of mushroom [1] contain mycotoxins, which, when ingested, could cause poisoning of varying degrees of severity and may even result in death. These poisonings can be classified according to 14 specific syndromes, some more serious than others: acromelalgic, cerebellar, coprinic, digestive (and resinoid), encephalopathy, gyromitrin, muscarinic, orellanus, pantherina, paxillus, phalloidin, proximien, psilocybin (or narcotic), and rhabdomyolysis syndrome [3,4]. In 2019, White et al. proposed a new classification of mycotoxic syndromes based on the main clinical signs rather than toxins. The new classification is made up of six groups (1. cytotoxic damage, 2. neurological damage, 3. muscular damage, 4. metabolic damage, 5. gastrointestinal irritation, and 6. other signs) divided into several subgroups [5]. Several case reports have shown that poisonings are mostly seasonal, between August and November, the period when mushrooms grow given the favorable climate [6]. In France, an average of 1300 poisoning cases per year was reported between 2010 and 2017 [6]. These poisonings are almost never documented by toxicological analysis, the cause of poisoning is mainly based on clinical signs and case history [7,8,9], since there are so few analytical methods for identifying the toxins described in the biological matrices [10,11]. There are many reasons: the numerous varieties of mushroom involved, mycotoxins with different chemical structures, a lack of knowledge about distribution and metabolism. The lack of analytical methods for identifying and quantifying these mycotoxins and their metabolites in the biological matrices is therefore an obstacle to knowledge and interpretation of cases of fatal and non-fatal poisoning. The main mycotoxins suspected in the most serious cases are as follows: orellanine, α- and β-amanitin, muscarine, muscimol, ibotenic acid, and gyromitrin. The aim of this work is to carry out a review of the literature, from an analytical point of view, of reported poisoning cases that involve these compounds, and to establish an inventory of the analytical techniques available for identifying and quantifying these mycotoxins.

## 2. Method

We performed a systematic review of the medical literature in order to identify manuscripts of interest. As the research was restricted to the forensic interest, our search strategies used a combination of standardized terms related to forensic situations (e.g., postmortem, intoxication, and poisoning) and key words that were implemented in NCBI PubMed (1900–present) and Google Scholar (1900–present). In order to reduce the number of results, the word “mushroom” was used as constant keyword. The used keywords were (number of identified articles): “orellanine” (50), “amanitins” (288), “ibotenic acid” (33), “muscimol” (44), “muscarine” (35), “gyromitrin” (27), “poisoning” (1906), and “intoxication” (266). Publications that were not found in the literature search but cited in retrieved publications were also considered. Overall, 256 cases reports were identified for orellanine, 800 for amanitins, 82 for ibotenic acid/muscimol/muscarine and at least 950 cases for gyromitrin. Focusing on the analytical concern, as we were interested in articles on identification and/or quantification of these mycotoxins in fungi or in human or animal biological matrices: additional key words were used in this way (e.g., chromatography, identification, quantification, etc.). All in all, 15 technical publications were selected for orellanine, 33 for the amanitins, 15 for ibotenic acid/muscimol, 8 for muscarine, and 7 for gyromitrin. Every reported concentrations data have been converted to international system units.

## 3. Orellanine

### 3.1. Toxic Compounds

**Figure 1:**
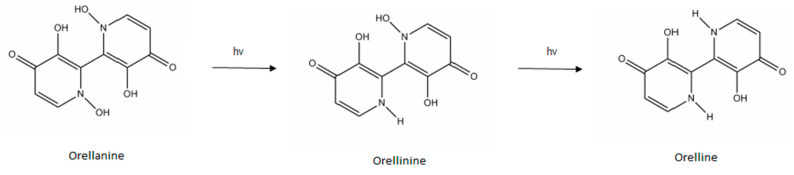
Structure of orellanine and its decomposition products.

Orellanine (C_10_H_8_N_2_O_6_, M = 252.2) was first identified in 1957 by Grzymala after a mass poisoning in Poland resulting in 19 deaths [12]. It was isolated in 1962 [13]. Orellanine is a bipyridine *N*-oxide (2,2′-bipyridine-3,3′,4,4′-tetrahydroxy-1,1′-dioxide) [14]. It is very polar (logP = −1.19) [15] and stable in the mushroom. However, it is photosensitive: once extracted, it is reduced by mono-hydroxylation to orellinine (C_10_H_8_N_2_O_5_, M = 236.2), which has the same toxic properties as orellanine, then by bi-dehydroxylation to orelline (non-toxic) [16] (Figure 1). Orellanine is not thermosensitive: cooking the mushrooms does not reduce their toxicity [16]. To the best of our knowledge, no metabolism data regarding orellanine has been reported in any publication.

### 3.2. Toxic Mechanism and Toxicity in Humans and/or Animals

The toxicity of orellanine lies in its strong nephrotic properties leading to acute renal failure (group 1C in the White et al. classification [5]). Its toxic mechanism has not been precisely established yet. However, Richard and his team have shown that orellanine is responsible for the inhibition of proteins in the cytoplasm and mitochondria of renal cells after tests on Madin–Darby canine renal cells [17]. Other hypotheses have been advanced such as the inhibition of DNA and RNA in the renal cells, glutathione depletion, or inhibition of mitochondrial adenosine triphosphate production [16,18].

There is high variability in clinical outcomes in the case of poisoning: the evolution can be spontaneously favorable or can deteriorate into chronic renal failure, requiring a kidney transplant [19]. There is no antidote for orellanine; treatment is symptomatic (hemodialysis, *N*-acetylcysteine, and steroids) [7,19,20]. Several studies in mice show that the oral median lethal dose (LD_50_) is between 30 and 90 mg/kg [21,22]. However, humans have been shown to be far more sensitive than mice to this mycotoxin. In practice, the ingestion of 6 mushrooms can lead to acute renal failure requiring dialysis [23].

### 3.3. Toxic Species

**Figure 2:**
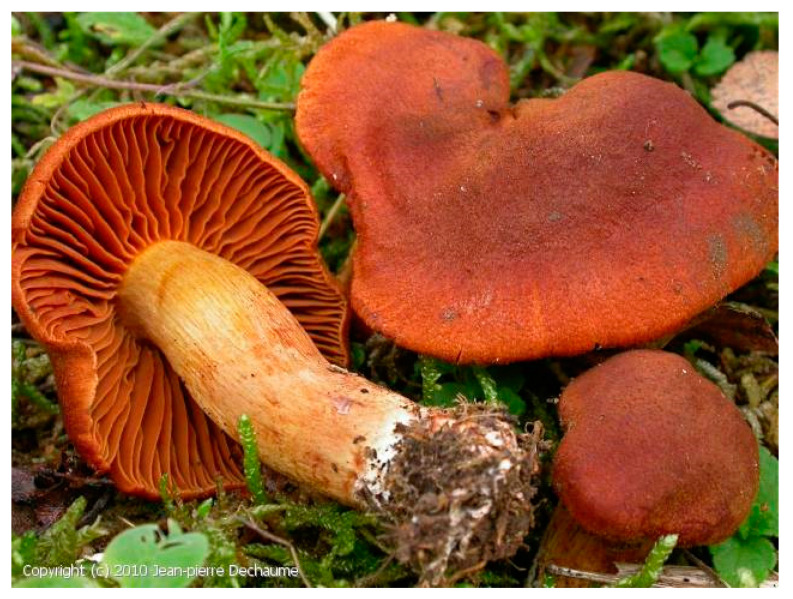
*Cortinarius orellanus* [30].

Orellanine is the main toxin found in mushrooms of the genus *Cortinarius* of the family Cortinariaceae. The most frequently reported in poisoning cases are *C. orellanus* [24,25] (Figure 2) and *C. speciosissimus* [7,19]. Some cases also mention *C. orellanosus* [23], *C. armillatus* [26], and *C. eartoxicus* [27]. The toxicity of *C. splendens* [28] is still in doubt. These species are mainly found in Europe and North America. Some cases of poisoning in Australia have also been reported [27,29].

### 3.4. Description of the Syndrome

Orellanine causes orellanus syndrome, which is characterized by a long latency period: between 2–4 and 14 days after ingestion [16]. To date, there is no scientific explanation for this exceptionally long latency period. The fact remains that this sometimes makes it difficult to link the ingestion with the clinical phase of poisoning. The first symptoms to appear are usually nausea, vomiting, diarrhea, stomach pains, extreme thirst, headaches, anuria, or polyuria depending on the case (cf. Table 1). These symptoms are followed by renal impairment necessitating transplantation. If left untreated, the patient may die of acute renal failure.

### 3.5. Human Poisoning Cases Reported

Many cases of orellanine poisoning have been reported in the literature since 1957. A number of them are listed nonexhaustively in Table 1. These cases include 27 reported deaths and 17 kidney transplants in people aged 14 and 60. Most poisonings are unintentional, sometimes by confusion with hallucinogenic mushrooms [29,31]. One case reports voluntary consumption of *Cortinarius orellanus* by a psychiatric patient [24]. Due to its long latency period, many patients consume mushrooms several times, sometimes a few days after the first meal [7,32,33]. The majority of patients have a serum creatinine over the physiological range at the arrival to the hospital. Those with a higher level underwent a renal transplantation.

### 3.6. Analytical Aspect

Research began in the late 1970s to develop a quick, sensitive, and reliable analytical method for identifying and quantifying orellanine in mushrooms as a first step, then in biological matrices such as blood, urine, or organs (cf. Table 2). Many methods are based on the thin layer chromatography, only one is based on the gas chromatography. Most recent methods consist of a liquid chromatography coupled with tandem mass spectrometry.

Many poisoning cases in the biological matrices documented by research for orellanine have revealed the absence of orellanine in urine, plasma, and dialysis fluids between 2 and 25 days after the ingestion of mushrooms [41]. However, Rapior et al. using thin layer chromatography coupled with spectrofluorometry, reported a concentration of 6.12 mg/L in plasma 10 days after the ingestion of mushrooms [24]. Orellanine has also been quantified several times in renal biopsies with concentrations between 35 and 3000 mg/L up to 180 days after poisoning [24,41].

## 4. α- and β-Amanitin

### 4.1. Toxic Compounds

**Figure 3:**
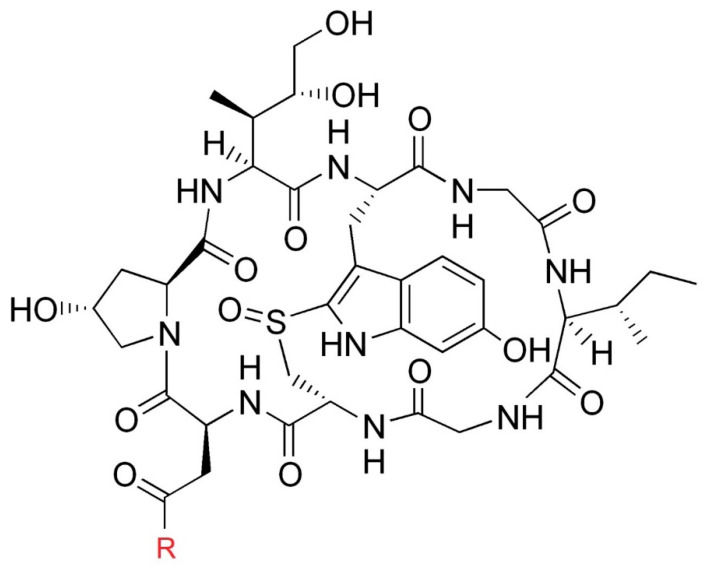
Structure of amatoxins. R = NH_2_ for α-amanitin, R= OH for β-amanitin.

Since the 1790s (Paulet’s research into the toxins of *Amanita phalloides*, 1793–1808) [57], researchers have taken an interest in the compounds responsible for the toxicity of *A. phalloides*. After the identification of other compounds contained in these mushrooms (e.g., phalloidin), Wieland et al. first isolated an amanitin in 1941 (which they later named α-amanitin) then 8 other amatoxins were isolated and their structure described (β-amanitin, γ-amanitin, ε-amanitin, amanin, amanullin, amaninamide, amanullinic acid, and proamanullin) [57]. The main toxins of certain mushrooms in this family are α-amanitin and β-amanitin. α-amanitin (C_39_H_54_N_10_O_14_S, M = 918.9) and β-amanitin (C_39_H_53_N_9_O_15_S, M = 919.9) are bicyclic octapeptides (Figure 3).

The amatoxins are not thermosensitive, which means they cannot be destroyed by either cooking or freezing the mushrooms [58]. Moreover, they are gastroresistant [58] and their metabolism is currently unknown.

### 4.2. Toxic Mechanism and Toxicity in Humans and/or Animals

In the new classification, the amatoxins are classified in the cytotoxic group (1A) [5] as they are responsible for inhibiting RNA polymerase II and the transcription of DNA into RNA by interfering with messenger RNA. This brings about inhibition of protein synthesis, which leads to cell necrosis. The first cells to be affected are those with a high rate of protein synthesis such as enterocytes, hepatocytes and proximal renal cells [59]. Studies in mice show that renal lesions only occur in poisoning with low levels of amatoxins. In poisoning cases with high levels, the subject die due to acute liver failure or hypoglycemia before the renal lesions appear [60,61]. Amatoxins are mainly eliminated in the bile, but there is an enterohepatic cycle, which prolongs the hepatoxic action [62].

Several studies show that the LD_50_ of α-amanitin in humans is estimated to be 0.1 mg/kg per os [63]. Bearing in mind that a sporophore of *Amanita phalloides* (20–25 g) can contain 5–8 mg of amatoxins [64], the ingestion of one *A. phalloides* mushroom is theoretically a lethal dose for a 75 kg man. The same order of magnitude is found in mice in a study published by Wieland in 1959 [57] (LD_50_ = 0.1 mg/kg for α-amanitin and 0.4 mg/kg for β-amanitin by intraperitoneal injection). Finally, it has been shown that the concentration of amatoxins in the mushroom increases during the first stages of the mushroom’s development, then decreases during the mature stage [65].

As with orellanine, no specific antidote exists for the amanitins. Treatment is symptomatic (dialysis, activated charcoal hemoperfusion, glucose/saline perfusion, etc.) [66,67]. Only kidney or liver transplantation (depending on the symptoms) can save a patient with multiple organ failure [67,68]. Some authors propose treatments such as thioctic acid (alpha lipoic acid) [69,70], penicillin G [71], or silibinin [72,73], which may be capable of limiting, if not inhibiting, the amatoxins’ penetration into the liver cells and/or interrupting the enterohepatic cycle of the toxins [74]. However, these treatments have not really been clinically proven and there is no evidence to support the use of penicillin G or of thioctic acid. They are therefore not considered as part of the protocol for treatment of amanitin poisoning.

In view of all the cases of amanitin poisoning reported in the literature, it seems clear that infants and small children are more sensitive to these mycotoxins than adults, probably because of their lower body mass: the same dose of toxins ingested will be more toxic and the percentage of fatalities will be higher in young subjects.

### 4.3. Toxic Species

**Figure 4:**
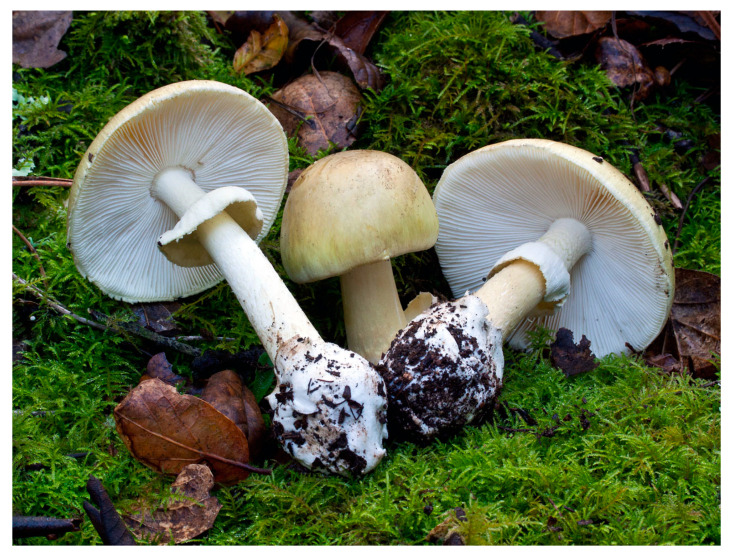
*Amanita phalloides* [79].

The amatoxins are the compounds responsible for the toxicity of *Amanita phalloides* [57] (Figure 4) also known as “death cap” in English-speaking countries [58], and without doubt the most well-known poisonous mushroom in the world. Probably all members of section Phalloideae contain potentially lethal levels of amanitins. These mycotoxins are also found in other species such as *A. verna* [75] and *A. virosa* [62], *A. bisporigera* [76], and *A. ocreata* [77]. Other genera contain amatoxins including *Galerina* (*G. marginata* and *G. autumnalis*) and *Lepiota* (*L. brunneoincarnata* and *L. helveola*) within the main species of concern [78].

Amatoxin-containing mushroom species have been worldwide identified (Northern, Central, and Western Europe, North and South America, South-East Asia, and Northern and Southern Africa) [58].

It should be noted that *Amanita phalloides* contains two other groups of toxins: phallotoxins and virotoxins [58]. These two families of cyclic peptides are only toxic by parenteral administration as they are hardly (or not at all) absorbed by the gastrointestinal tract [58]. They are therefore not usually taken into consideration in *Amanita phalloides* poisoning.

### 4.4. Description of the Syndrome

The amatoxins are responsible for phalloidin syndrome, which, like orellanus syndrome, is characterized by a long latency period (between 6 and 24 h) after ingestion of the mushroom [58]. First occurring symptoms are gastrointestinal (nausea, vomiting, diarrhea, and stomach pains) over a period of about 24 h. The second stage is a period of remission, usually lasting 24–36 h. During this period, the serum activity levels of aspartate aminotransferase (AST) and alanine aminotransferase (ALT) rise progressively, showing liver damage. The third stage is characterized by renal and hepatic impairment, which could result in hepatic encephalopathy, convulsions, coma and death (4–7 days after ingestion of mushrooms) [74]. Death by amatoxin poisoning is most often caused by liver, or kidney failure, or sometimes both (cf. Table 3).

### 4.5. Human Poisoning Cases Reported

Given the large number of mushroom species containing amanitins throughout the world, a great number of amatoxin poisoning cases have been reported in the literature since the beginning of the last century (Table 3). Of these recorded poisonings, 72 deaths and 33 liver transplants are listed. Five of the deaths occurred up to several months after liver transplantation. This suggests persistent toxicity capable of damaging the graft. One case is unusual, the patient ate 2 caps of *Amanita phalloides* only in order to test the toxicity [114].

The result is fatal in 10–30% of cases [58], with the percentage tending to decrease mainly due to liver transplantation.

### 4.6. Analytical Aspect

Research began in the mid-1970s to develop a sensitive and reliable analytical method for identifying and quantifying α- and β-amanitin through radioimmunological techniques, thin layer chromatography or liquid chromatography-UV detection. Technological developments over the years have enabled researchers to reach better and better sensitivity levels using high-resolution mass detectors (cf. Table 4).

Testing for amanitins in various biological samples in a known case of amatoxin poisoning has revealed the elimination kinetics of these compounds. It is possible to find amanitins in blood (plasma or serum) up to 36–48 h after ingestion [61,90,151] in concentrations varying from 10 to 200 µg/L [91] and in urine up to 96 h after ingestion [89,151]. The urine concentrations range from 1 to 7100 µg/L, with a peak between 24 and 72 h [90,140,151].

Jaeger et al. have shown that it is also possible to find high concentrations of α- and β-amanitin in gastroduodenal fluid and feces (between 208 and 4950 µg/L in gastroduodenal fluid and between 23 and 14900 µg/L in feces) [90].

The amanitins have hepatic and renal tropism. As a consequence, it should be of interest to assay them in these matrices. Jaeger et al. reported concentrations of 10–3298 µg/L found in the liver and kidney samples (from autopsy or biopsy) of poisoned subjects [90].

There is an immunological technique for assaying alpha and gamma amanitins (but not beta amanitin) in urine available as a kit (BÜHLMANN ELISA kit). Its limit of detection is 0.22 µg/L with a limit of quantification of 1.5 µg/L [152].

## 5. Muscarine

### 5.1. Toxic Compounds

**Figure 5:**
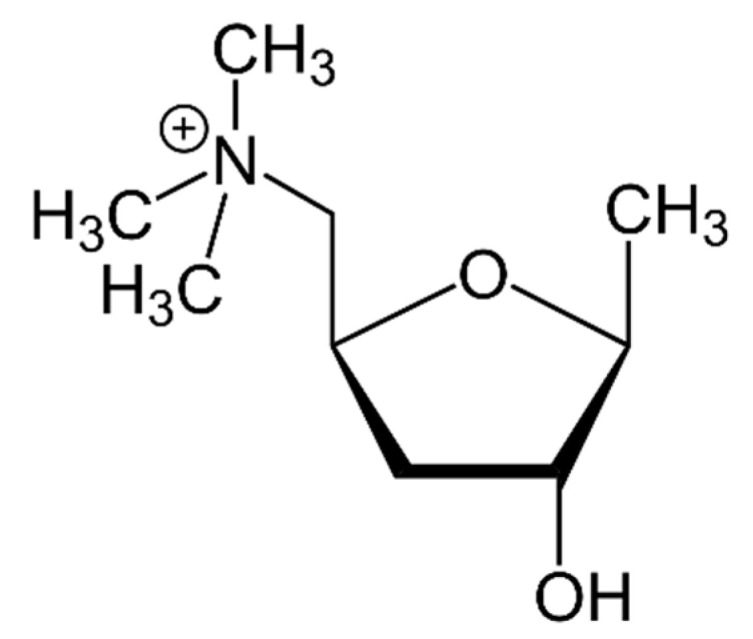
Structure of muscarine.

The first attempt to isolate muscarine, which was considered the main active substance in *Amanita muscaria* [153], dates back to the early 1810s with Braconnot and Schrader. At that time several researchers had tried in vain to isolate this psychoactive compound. It was not until 1869 that Schmiedeberg and Koppe believed they had isolated the substance and called it muscarine. The substance they isolated proved to be a mixture of muscarine and choline. Pure muscarine was actually isolated for the first time by King in 1922 [154]. The structure of muscarine was proposed in 1957 by Kögl et al. [155]: C_9_H_20_NO_2_^+^, M = 174.3 (Figure 5). Muscarine (tetrahydro-4-hydroxy-*N,N,N*-5-tetramethyl-2-furanmethanaminium) is a water-soluble thermostable alkaloid [154]. To the best of our knowledge, no studies or metabolism data have been published about this mycotoxin.

### 5.2. Toxic Mechanism and Toxicity in Humans and/or Animals

Muscarine is an agonist for the neurotransmitter acetylcholine; it activates muscarinic acetylcholine receptors and thereby activates the parasympathetic nervous system [155]. Due to its positively charged quaternary amine group, muscarine does not cross the blood–brain barrier and therefore does not reach the central nervous system. This mechanism of action puts it in group 2B of the White et al. classification [5] (neurotoxic molecules that do not reach the central nervous system). Unlike many mycotoxins, there is an antidote to muscarine: atropine. Administered intravenously, atropine counters the toxic cardiac effects of muscarine [156]. Muscarine poisoning must be proven (for example by identifying the mushroom species ingested) before administering atropine, since atropine can exacerbate some symptoms if administered in error (see ibotenic acid and muscimol, below).

The toxic effects of muscarine vary according to the amount ingested. Muscarine poisoning is rarely fatal; patients with pre-existing cardiac disorders will be more sensitive. The symptomatology usually resolves after a few hours. In cases where the patient is severely dehydrated, compensation for fluid and electrolyte loss should be considered [2].

Toxicity studies show the i.v. LD_50_ of muscarine in mice is 0.23 mg/kg [157,158]. No numerical data for humans have been published.

No mechanism or preferential route of elimination of muscarine from the organism has been described in the literature.

### 5.3. Toxic Species

**Figure 6:**
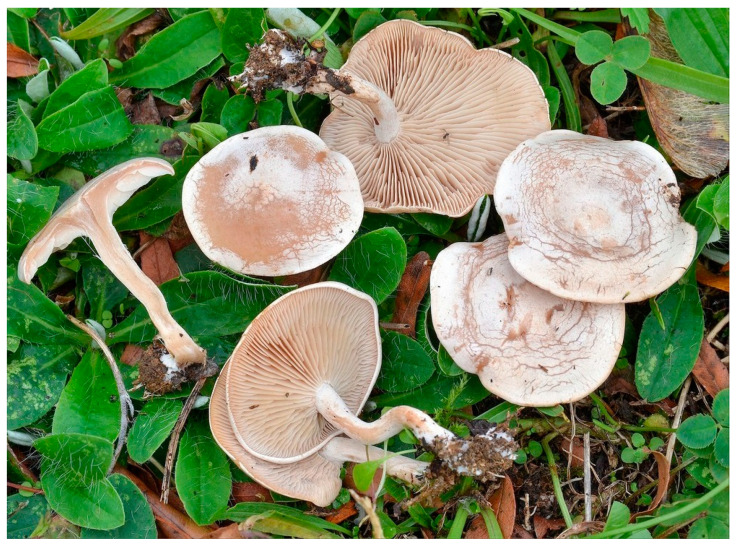
*Clitocybe rivulosa* (copyright ©Andgelo Mombert) [161].

Muscarine is actively present in several mushroom families: around 40 *Inocybes* of the family Inocybaceae (*I. erubescens, I. subdestricta, I. fastigiata, I. geophilla*, etc.), around 15 *Clytocybes* (Figure 6) of the family Tricholomataceae (*C. cerussata, C. dealbata, C. rivulosa, C. phylophilla*, etc.) [156]. It is also found in the genus *Amanita* (*A. muscaria* and *A. pantherina*) but in minute quantities [159], which makes its toxic action insignificant compared with these mushrooms’ other active compounds. *Amanita muscaria* takes its name from muscarine since, as explained above, muscarine was isolated from this species. However, the fly agaric only contains 0.0002–0.0003% of muscarine [153,159,160]. By comparison, *I. subdestricta* contains 0.43% and *C. dealbata* 0.15% [153].

Due to the great diversity of mushrooms containing muscarine, the toxin has been identified on every continent.

### 5.4. Description of the Syndrome

The syndrome associated with muscarine is called muscarinic syndrome. It has a short latency period (<6 h) as the first symptoms appear between 15 min and 2 h after ingestion [156]. The main clinical signs of muscarine poisoning are gastrointestinal distress (nausea, vomiting, diarrhea, and stomach pains), extreme sweating, bronchial, salivary and ocular hypersecretion, and blurred vision. Observed bradycardia, hypotension, and miosis are the direct consequences of acetylcholine receptors activation. In the most severe cases muscarine can cause myoclonus, convulsions, and loss of consciousness that may lead to coma and the death of the patient (cf. Table 5).

### 5.5. Human Poisoning Cases Reported

Case reports about muscarine poisoning are relatively rare. Table 5 shows published cases of muscarine poisoning. A fatal outcome was observed in three cases: an 11-year-old child [174], a 67-year-old woman presenting comorbidities (diabetes, arterial hypertension, and respiratory insufficiency) [172], and a 53-year-old woman with no particular medical history [170]. The other cases present a positive outcome.

### 5.6. Analytical Aspect

Since muscarine was isolated in 1922 [154], few analytical techniques have been published for identifying and quantifying the compound in different matrices. The first published techniques used thin layer chromatography or gas chromatography with mass detection for qualitative and/or quantitative analysis of muscarine in mushrooms. The technological advances of the early 21st century have enabled considerably greater sensitivity with liquid chromatography techniques coupled to tandem mass spectrometry. With these techniques it is now possible to quantify muscarine in biological matrices such as urine (Table 6).

To the best of our knowledge, no research on muscarine in blood or any other biological matrix has been published. Only one publication mentions a numerical value for muscarine in urine: 0.045 mg/L of muscarine was found in the urine of a 55-year-old suspected of having ingested *A. muscaria* [155].

## 6. Ibotenic Acid, Muscimol

### 6.1. Toxic Compounds

**Figure 7:**
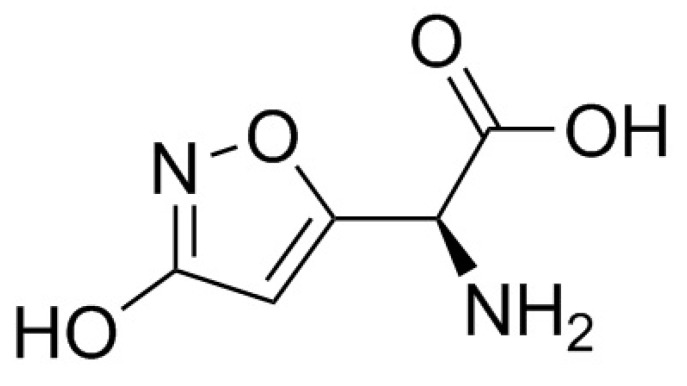
Structure of ibotenic acid.

**Figure 8:**
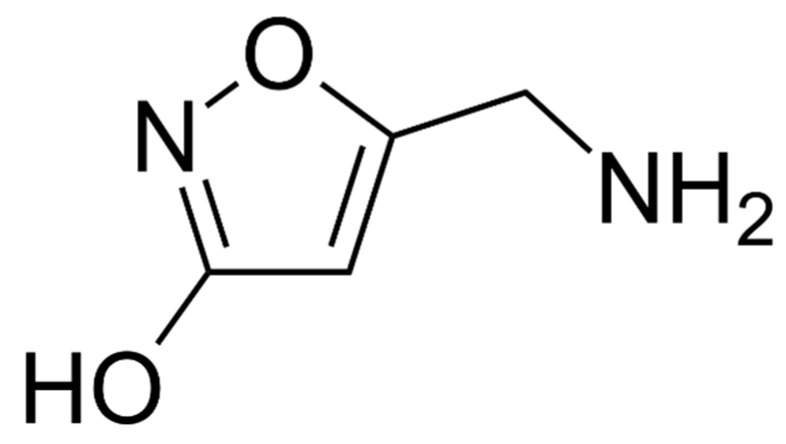
Structure of muscimol.

Ibotenic acid or α-amino-3-hydroxy-5-isoxazoleacetic acid (C_5_H_6_N_2_O_4_, M = 158.1) is an alkaloid, which is degraded by decarboxylation into muscimol (3-hydroxy-5-aminomethylisoxazole, C_4_H_6_N_2_O_2_, M = 114.1; Figure 7 and Figure 8). These compounds, isolated and described in the 1960s by a Japanese team, are thermostable [153] but the dehydration of ibotenic acid leads to the formation of muscimol by decarboxylation [183]. It would therefore be logical to consider the toxicity of cooked *A. muscaria* and *A. pantherina* mushrooms to be mainly attributable to muscimol. These two mycotoxins are the major factors in poisoning, but other toxins have also been identified in the mushrooms, including muscarine, in very low quantities, and muscazone, a structural isomer of ibotenic acid with less potent psychoactive properties than muscimol or ibotenic acid [153,183].

DeFeudis [160] states that muscimol is metabolized quickly after ingestion, and that consequently, its toxicity is shared with its psychoactive metabolites. However, no concrete metabolic study has been published about muscimol or ibotenic acid.

### 6.2. Toxic Mechanism and Toxicity in Humans and/or Animals

Ibotenic acid and muscimol are isoxazoles derived from glutamic acid and γ-aminobutyric acid (GABA) respectively [183]. Ibotenic acid and muscimol can cross the blood–brain barrier and thus act on the central nervous system [184], which puts them in group 2C of the White et al. classification [5] (neurotoxic molecules that reach the central nervous system). Ibotenic acid is a glutamate neurotransmitter agonist, a powerful neuronal excitant. It acts on the glutamic acid receptors associated with memory and learning. Muscimol is a γ-aminobutyric acid (GABA) agonist. It acts on the GABA receptors with a depressant effect and therefore causes related toxic effects such as visual distortions/hallucinations, loss of balance, slight muscle contractions, and altered sensory perceptions [153,183]. These two alkaloids are preferentially eliminated in urine [153,183]. Ibotenic acid and muscimol can be detected in urine one hour after mushroom ingestion [153].

Fatal poisoning by ibotenic acid and muscimol is very rare [153]. There is no antidote; the only treatment is symptomatic. Hospitalization for neurological surveillance is recommended [156]. In some cases it is necessary to sedate the patient to manage excessive agitation [9,162]. Atropine is to be avoided as it has a similar action to ibotenic acid and muscimol.

Ibotenic acid and muscimol are lethal in very high doses. The LD_50_ in rats is 129 mg/kg for ibotenic acid and 45 mg/kg for muscimol [158,185,186]. Stebelska [185] refers to a study of the toxicity of isoxazoles on mammals: the oral LD_50_ for muscimol is 10 mg/kg in rabbits and the oral LD_50_ for ibotenic acid is 38 mg/kg in mice. As with muscarine, no data for humans have yet been published.

A sporophore of *Amanita muscaria* can contain between 292 and 6570 µg/g of ibotenic acid and between 73 and 2440 µg/g of muscimol [187]. Given the average weight of 60 g and the minimal dose to produce psychotropic effects of 30–60 mg of ibotenic acid and around 6–10 mg of muscimol, a single mushroom is enough to experience hallucinogenic effects [185]. Some studies have shown that the intensity of the effects varies according to which part of the mushroom is consumed. Indeed, the cap of the mushroom has a higher concentration of psychoactive substances than the stem [188,189].

### 6.3. Toxic Species

**Figure 9:**
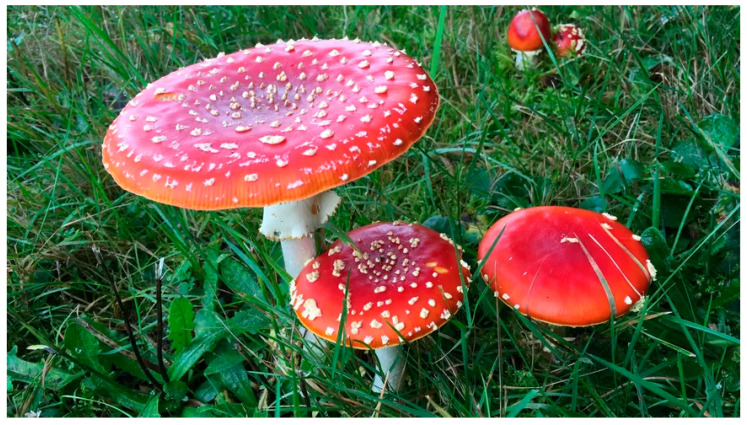
*Amanita muscaria* [190].

Ibotenic acid and muscimol are mainly found in *Amanita muscaria* (Figure 9) and *Amanita pantherina* mushrooms, which belong to the genus *Amanita* of the family Amanitaceae. Virtually all mushrooms in genus *Amanita* contain high levels of muscimol and ibotenic acid. *A. muscaria* is undoubtedly the most iconic mushroom in the world, represented in illustrations, cartoons, etc., due to its bright colors and white spotted cap. These mushrooms have been identified in the United States, sub-Saharan Africa (South Africa, Zimbabwe) Japan, and Europe (cf. Table 5).

The possession, purchase, and sale of ibotenic acid and muscimol are not regulated in France. However, the possession, purchase, and sale of *Amanita muscaria* are illegal in the Netherlands [191], the state of Louisiana in the USA, the UK [192], and Romania [192]. In Thailand hallucinogenic mushrooms are classified as class V narcotics and are therefore illegal [193]. In Japan these two mushroom species are sold openly as dried mushrooms or dried mushroom “powder” on the internet and in “smoke shops” [186].

### 6.4. Description of the Syndrome

The syndrome produced by consuming mushrooms containing ibotenic acid and muscimol is called pantherina syndrome (or myco-atropine syndrome) [156]. The syndrome is characterized by a short latency period (30 min to 3 h) [156]. The first perceptible effects after ingestion are mainly nausea, vomiting, and diarrhea, followed by characteristic symptoms of central nervous system dysfunction (confusion, dizziness, myoclonus, visual and auditory hypersensitivity, and distortion of time and space) accompanied by mydriasis, fatigue, and drowsiness (cf. Table 5). The phenomenon of hallucinations has been discussed. After 2 h the subject presents altered states of consciousness lasting approximately 8 h [153].

Pantherina syndrome is sometimes confused with drunkenness.

### 6.5. Human Poisoning Cases Reported

The consumption of *Amanita muscaria* is connected with mysticism since the mushroom’s psychotropic properties have been known and prized for several thousand years. *A. muscaria* was traditionally used in religious, spiritual, or shamanic rituals by some tribes in Northern Europe and Northern Asia (Siberian shamans of tribes such as the Ostyak, Vogul, Kamchadal, Koryak, and Chukchi) [153]. The “Rig Veda”, the ancient Hindu text considered one of the world’s great religious works (composition estimated between 1500 and 900 BC) [194], advocates “Soma”. The term Soma has several meanings in Hindu mythology: a ritual drink, the plant (or the mushroom), and the god. Several hypotheses argue that Soma was extracted from *Amanita muscaria* [195,196]. In his book “*Amanita muscaria*; Herb of Immortality” Teeter considers the fly agaric to be at the centre of all religions and beliefs [197]. Theories about *A. muscaria* as soma have been very thoroughly debunked [198].

*A. muscaria* or *A. pantherina* poisonings can happen accidentally, through confusion with an edible mushroom species or ignorance of the fungi kingdom. However, a large proportion of these poisonings are from voluntary recreational consumption from those seeking psychotropic effects. Table 5 lists some examples. Only one case of death of a 55-year-old man attributed to an *Amanita muscaria* poisoning was reported [155]. Unfortunately, in this case, only muscarine in urine was quantified, neither ibotenic acid nor muscimol.

### 6.6. Analytical Aspect

Analytical techniques have been developed since the early 1980s with the aim of identifying and quantifying the principal mycotoxins responsible for pantherina syndrome. Liquid chromatography is the most widely used technique. It was not until the late 2000s that researchers considered the detection of isoxazoles in biological matrices (urine and serum; Table 7).

Some poisoning cases have been documented where patients’ biological samples were investigated for ibotenic acid and muscimol. Stříbrný et al. [176] reported varying concentrations of ibotenic acid between 32 and 55 mg/L, and of muscimol between 6 and 10 mg/L in urine (3–8 h after ingestion). Hasegawa et al. [177] reported concentrations of 96 µg/L of ibotenic acid and 101 µg/L of muscimol in the serum of a subject poisoned by *A. ibotengutake* (without specifying the period between ingestion and sampling).

## 7. Gyromitrin

### 7.1. Toxic Compounds

**Figure 10:**
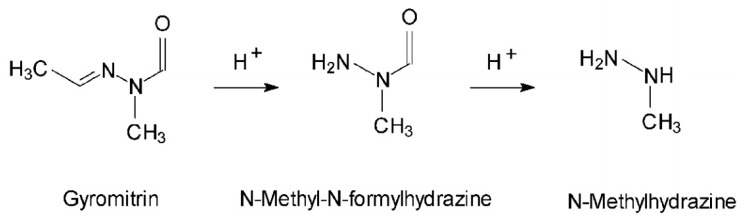
Structure of gyromitrin and its metabolites [209].

In 1885, Boehm and Külz isolated an oily substance from the false morel, which they believed to be the substance responsible for the mushroom’s toxicity. More advanced studies have shown that it is actually a mixture of non-toxic organic acids. Gyromitrin was finally isolated, synthesized and definitively identified in 1968 by List and Luft as acetaldehyde *N*-methyl-*N*-formylhydrazone or gyromitrin (C_4_H_8_N_2_O, M = 100.1) [206,207,208]. The hydrolytic cleavage of gyromitrin (Figure 10) leads to the formation of *N*-methyl-*N*-formylhydrazine and then methylhydrazine (or monomethylhydrazine, MMH) [209,210], which is used in astronautics as a rocket propellant [209]. Gyromitrin belongs to the hydrazine family and is volatile, thermosensitive, and very soluble in water [207]. This mycotoxin can be partially eliminated by drying or boiling the mushroom. Pyysalo [211] has shown that these measures can reduce the quantity of gyromitrin originally contained in the mushroom by up to 99–100%.

### 7.2. Toxic Mechanism and Toxicity in Humans and/or Animals

Gyromitrin is classed as a GABA-inhibiting mycotoxin, group 4A in the White et al. classification [5]. Its mechanism of toxic action is connected with the production of MMH. MMH interacts with pyridoxine dependent coenzymes, resulting in inhibition of glutamic acid decarboxylase and thus reduced GABA production, causing the neurological symptoms to occur. MMH can also cause methemoglobinemia [207,212]. In addition, MMH produces radical species that lead secondarily to hepatic cytolysis [207].

*N*-methyl-*N*-formylhydrazone and methylhydrazine are known to be hepatotoxic through the mechanism of producing radical species, but they are also known to be carcinogenic in animals [209,213].

Several studies have been conducted on animals to determine the lethal dose of 50% for gyromitrin and MMH. Patocka et al. [209] reported an oral LD_50_ for gyromitrin of 344 mg/kg in mice, 320 mg/kg in rats, 50–70 mg/kg in rabbits, and a resistance of over 400 mg/kg in chickens. In humans, the oral LD_50_ is estimated at 20–50 mg/kg in adults and 10–30 mg/kg in children [207]. Studies of the lethal dose of monomethylhydrazine have also been published, reporting a dose of 4.8–8 mg/kg in adults and 1.6–4.8 mg/kg in children [212]. Pyysalo et al. reported a concentration of 50 mg of gyromitrin/kg in fresh mushrooms (Finnish species).

There is considerable variation in individual responses to gyromitrin poisoning: ranging from simple stomach upset to the death of the patient (cf. Table 8). The outcome is fatal in approximately 10% of cases [207].

Treatment of gyromitrin poisoning is symptomatic. It may include administration of vitamin B6 (pyridoxine) to stop seizures and/or anticonvulsants such as clonazepam [207,212].

### 7.3. Toxic Species

**Figure 11:**
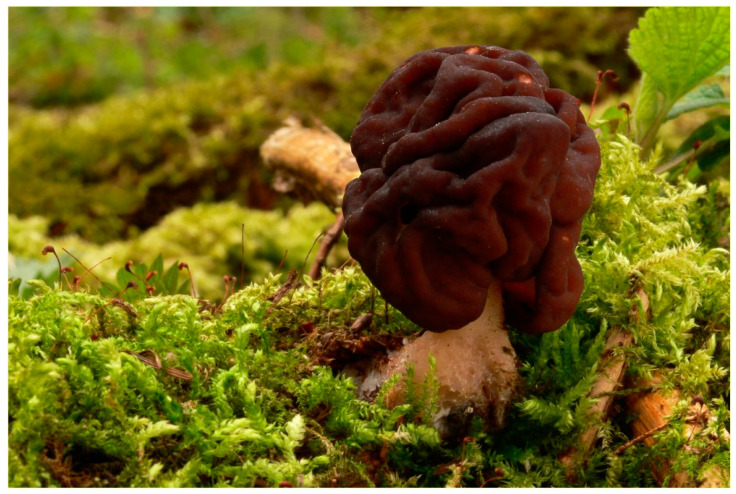
*Gyromitra esculenta* [218].

Gyromitrin is the main toxin in mushrooms of the genus *Gyromitra* of the family Discinaceae. The most common mushroom is *Gyromitra esculenta* (Figure 11), which is often confused with morel, hence its nickname: false morel [207] shares a subgroup with *G. fastigiate* [207] and G. ambigua [217]. There is no evidence that *G. gigas* contains gyromitrin. It would appear that a large proportion of the genus *Gyromitra* contains gyromitrin [209].

It should be noted that *G. esculenta* contains other toxins beside gyromitrin: pentanal *N*-methyl-*N*-formylhydrazone, 3-methylbutanal *N*-methyl-*N*-formylhydrazone, and hexanal *N*-methyl-*N*-formylhydrazone [210]. All these compounds lead to the formation of methylhydrazine by hydrolysis [209,210]. In addition, there is a small amount of *N*-methyl-*N*-formylhydrazine in the mushroom, formed by hydrolytic cleavage [209].

This fungi genus is found mainly in the northern hemisphere (Canada, United States, and Eastern Europe). Long considered edible, *G. esculenta* has been the cause of many deaths.

### 7.4. Description of the Syndrome

The syndrome resulting from gyromitrin poisoning is called gyromitra syndrome [156]. It is characterized by a long latency period (between 5 and 12 h) after consuming the mushrooms [207]. Like the majority of mushroom poisonings, the first perceptible symptoms are nausea, vomiting, stomach pains, and sometimes bloody diarrhea, resulting in dehydration and headaches. MMH being hepatotoxic, there is often jaundice, indicating liver damage. In severe cases of poisoning there are altered states of consciousness, lack of motor coordination, seizures, and coma, which may lead to the death of the patient (c.f. Table 8).

In most cases the symptoms disappear 2–6 h after ingesting the mushrooms [212].

### 7.5. Human Poisoning Cases Reported

The first cases of gyromitrin poisoning were reported in 1782, then towards the end of the 1800s [215,216]. Franke et al. [215] reported a large number of poisonings in Eastern Europe between 1782 and 1965. However, there are fewer cases of poisoning reported than for the other mycotoxins due to this toxin’s thermosensitivity (Table 8). Due to the long latency period, some patient ate mushrooms several times. Some of these patients died of liver failure [216].

### 7.6. Analytical Aspect

Very few quantitative analytical techniques regarding gyromitrin have been reported in the literature (Table 9). The majority report a quantification of MMH in mushrooms using gas chromatography. Only three publications have covered biological matrices in mice or humans. It should be noted that some authors measure methylhydrazine rather than gyromitrin because of its rapid metabolization in vivo. To our knowledge, no technique using liquid chromatography to identify and quantify gyromitrine or its metabolites was published.

No data have been published to date on the quantification of gyromitrin in human biological matrices following *G. esculenta* poisoning.

## 8. Conclusions

This review of the literature took an analytical perspective, and focused on highly toxic mycotoxins (orellanine, α- and β-amanitin, muscarine, ibotenic acid, muscimol, and gyromitrin). It identifies a set of knowledge gaps. There is indeed a lack of scientific data, particularly regarding the metabolism of mycotoxins in biological matrices, but there is also a lack of analytical tools. There is a real need for the development and validation of specialized analytical methods adapted for the analysis of these mycotoxins in various matrices. Their implementation in the context of a clinico-biological study comparing the results of biological samples analysis (identification and assay) with the case history and clinical signs of confirmed or suspected poisoning victims could strengthen our understanding and treatment of these poisonings.

## Figures and Tables

**Table 1:** Cases of orellanine poisoning.

Ref.	Date of Intoxication	Country	*N*	Sex/Age	Offset of Symptoms/Delay before Hospitalization	Symptoms	Treatment	Notes	Toxin Quantification	Outcome	Mushroom Species
[12]	1955–1957	Poland	144	-	-	-	-	-	-	25 deaths	*Cortinarius orellanus*
[34]	-	Finland	9	-	-		6 hemodialysis	-	-	4 renal transplantation	*Cortinarius speciosissimus*
[34]	NC	Sweden	2	M/24	NC/NC	Nausea, vomiting, abdominal pain	Gastric aspiration, hemoperfusion, hemodialysis	-	-	Renal function normal	*Cortinarius speciosissimus*
				F/47	NC/NC	Nausea, abdominal pain		-	-	Renal function normal	
[19]	August 1979	Scotland	3	M/31	H 36/D 10	Nausea, vomiting, anorexia, muscle and abdominal pain, night sweats, headache, bilateral loin pain, severe burning thirst, oliguria, anuria, acute renal failure	Peritoneal dialysis, hemodialysis	Plasma creatinine: 2945 µmol/L at D 10; Plasma urea: 48 mmol/L at D 10; Percutaneous renal biopsy at W 3 and W 7 after admission	-	Renal transplantation at Mo 9	*Cortinarius speciosissimus*
				M/30	NC/NC		Hemodialysis	Consumption of the same mushroom on 2 consecutive days; Plasma creatinine: 1925 µmol/L at D 10; Plasma urea: 42 mmol/L at D 10; Percutaneous renal biopsy at W 2^1/2^ and W 6 after admission	-		
				F/25	D 2/D 11		-	Consumption of the same mushroom on 2 occasions; Plasma creatinine: 91 µmol/L at D 11; Plasma urea: 4.5 mmol/L at D 11	-	Renal function normal	
[35]	1981	France	5	-	-	-	-	-	-	3 positive development; 1 death of intracerebral hematoma; 1 chronic renal failure	*Cortinarius splendens*
[36]	September 1981	Italy	2	M/38	D 2/NC	Gastrointestinal disorder, acute renal failure	Plasma exchange, dialysis	Renal biopsy reveal tubulointerstitial necrosis + interstitial oedema	-	Positive development	*Cortinarius speciosissimus*
				F/38						Renal failure for 6 months	
[37]	NC	Germany	2	-	-	-	-	-	-	2 renal failure	*Cortinarius speciosissimus*
[32,33]	1979–1993	Sweden	22	M/41	D 1/D 8	Vomiting, severe burning thirst, polyuria, oliguria	Hemoperfusion, hemodialysis, peritoneal dialysis	3 meals during 2 weeks; Serum creatinine: 1600 µmol/L at D 8	-	Renal transplantation at Y 3	*Cortinarius speciosissimus*
				M/44	D 2/D 10	Nausea, vomiting, abdominal pain, oliguria, acute renal failure	Peritoneal dialysis, hemodialysis	Serum creatinine: 1500 µmol/L at D 10; Uremia: 37 mmol/L at D 10; Renal biopsy at Mo 2 reveal normal glomeruli and atrophic tubuli	-	Renal transplantation at Mo 9–10	
				F/47	D 4/D 5	Nausea, vomiting, abdominal and muscular pain, intense burning thirst, polyuria,	Hemoperfusion, hemodialysis	Consumption of 15 fruit bodies; Serum creatinine: 402 µmol/L at D 5, 780 µmol/L at D 12	-	Renal function normal	
				M/24	D 4/D 5 after 1st meal	Nausea, abdominal and muscular pain, heavy thirst	Hemoperfusion, hemodialysis	Consumption of 4–6 fruit bodies on 2 occasions; Serum creatinine: 158 µmol/L at D 5, 380 µmol/L at D 12	-	Renal function normal	
				F/60	H 12/NC	Nausea, vomiting, hematuria, proteinuria, glycosuria, anuria	Hemoperfusion, hemodialysis	Consumption of 7 mushrooms; Serum creatinine: 154 µmol/L at D		Renal transplantation at Mo 6	
				M/21	D 3/NC	Polyuria and then anuria	Hemoperfusion, hemodialysis	Consumption of 3 mushrooms	-	Renal transplantation at Mo 30; Renal biopsy on transplantation kidney at Y 7 reveal atrophic tubuli	
				M/14	D 4/D 10	Nausea, vomiting	Peritoneal dialysis	Serum creatinine: 1350 µmol/L at D 10; Uremia: 68 mmol/L at D 10		Renal transplantation at Mo 8	
[38]	NC	Switzerland	1	M/14	NC/D 5	Vomiting, anorexia, renal pain, leukocyturia, hematuria	hemodialysis		-	Renal transplantation at Mo 14	*Cortinarius speciosissimus*
[24]	November 1987	France	1	F/31	D 8/D 10	Nausea, vomiting, severe thirst, abdominal pain, renal failure	Hemodialysis, hemodialysis resin, plasmapheresis resin, furosemide, diltiazem, dopamine, vitamin C, amino acid	Psychiatric patient; Deliberate ingestion of 2 fruit bodies (≈ 20 g); Serum creatinine: 1100 µmol/L at D 10; Renal biopsy at D 13 and 180 reveal chronic interstitial nephritis	Detection by TLC; Plasma at D 10 = 6.12 mg/L; Renal biopsy at D 13 ≈ 280 mg/L, at D 180 = 3000 mg/L	NC	*Cortinarius orellanus*
[25]	September 1987	France	26	M/between 21 and 28	D 2–9/D 10–12	Digestive disorders, asthenia, thirst, headache, chills, polyuria, lumbar pain, paresthesia, dysgeusia, skin rash, 12 acute tubulointerstitial nephritis with acute renal failure	8 hemodialysis; 9 under corticosteroids	During a survival exercise; Serum creatinine: 172–2248 µmol/L	-	1 renal transplantation at Mo 10; 1 chronic hemodialysis; 2 persisting renal failure; 22 renal function normal	*Cortinarius orellanus*
[39]	NC	Canada	1	F/20	H 8/D 5	Nausea, vomiting, diarrhea, abdominal pain, proteinuria, pyuria, hematuria	Sodium polystyrene sulfonate	Confusion with hallucinogenic mushrooms; Serum creatinine: 356 µmol/L at D 5; Uremia: 10.1 mmol/L at D 5	-	Renal function normal	NC
[40]	NC	Germany	1	M/27	D 9/D 14	Nausea, anorexia, oliguria, leukocyturia, acute renal failure	Hemodialysis, peritoneal dialysis	Serum creatinine: 1450 µmol/L at D 14; Uremia: 59 mmol/L at D 14; Renal biopsy at D 14 reveal tubulointerstitial nephritis	-	Renal transplantation	*Cortinarius orellanus*
[41]	1994–1995	Austria/Northern Italy	8	M/74	D 2/NC	Nausea, abdominal and loin pain, uremia	dialysis	-	TLC on fluids failed to detect orellanin	NC	*Cortinarius speciosissimus*
				F/33	D 2/NC			Renal biopsy at D 10	Detection of orellanin in renal biopsy at D 10 by TLC ≈ 160 mg/L		
				F/34	D 4/NC			-	TLC on fluids failed to detect orellanin		
				M/43	D 4 /NC			-			
				M/59	D 5/NC			-			
				F/52	D 3/NC			-			
				M/82	D 5/NC			-			
				M/54	D 5/NC			-			
[41,42]	August 1995	Austria	1	M/23	NC/D 14	Nausea, abdominal and loin pain, acute anuria	Hemodialysis	Consumption of 5 raw fruit bodies confused with hallucinogenic mushrooms; Renal biopsy at D 180 reveal acute interstitial nephritis	Orellanin not detected in the renal biopsy	Peritoneal dialysis; Waiting for renal transplantation 6 months later	*Cortinarius speciosissimus*
[41,43]	NC	Austria	1	M/28	D 7/D 21	Nausea, vomiting, lumbar pain, proteinuria, leukocyturia, erythocyturia, hyperphosphatemia, dehydration, anuria	Hemodialysis, probucol	Consumption of 2 raw fruit bodies confused with hallucinogenic mushrooms; Serum creatinine: 2033 µmol/L at D 16; Uremia: 28.3 mmol/L at D 16	Detection of orellanin in renal biopsy at W 5 by TLC ≈ 35 mg/L	Hemodialysis 12 months later; Waiting for renal transplantation	*Cortinarius speciosissimus*
[44]	NC	Austria	4	M/37	NC/NC	Nausea, vomiting, dizziness, oliguria	hemodialysis	Serum creatinine: 813 µmol/L at D 14; Uremia: 47 mmol/L at D 14	-	Positive development	*-*
				F/78	D 7/D 11	Nausea, vomiting, dizziness, malaise, arthralgia, severe metabolic acidosis, anuria	Isradipine, urapidil, clonidine, hemodialysis, steroids	Serum creatinine: 1768 µmol/L at D 11; Uremia: 80 mmol/L at D 11; Kidney biopsy reveal acute tubular necrosis, interstitial fibrosis	-	Chronic hemodialysis 10 months later	
				F/56	D7/not admitted to the hospital	Nausea, vomiting, malaise	NA	-	NA	Renal function normal	
				M/70	NC/D 9	Nausea, vomiting, anuria, malaise, arthralgia	hemodialysis	Underwent partial gastrectomy in 1949; Serum creatinine: 1768 µmol/L at D 9; Uremia: 48.3 mmol/L at D 9	-	Chronic hemodialysis 10 months later	
[31]	NC	Spain	1	M/32	D 5/D 15	Nausea, vomiting, anorexia, flanks and abdominal pain, acute renal failure, insomnia, anuria, dehydration, leukocytosis, glycosuria, proteinuria	Hemodialysis, rehydration	Past of drug addict; Voluntary ingestion of 2 fruits bodies looking for hallucinogenic effects; Serum creatinine: 477 µmol/L at D 15; Uremia: 8.2 mmol/L at D 15; Renal biopsy at D 16 reveal acute tubulointerstitial nephritis	-	Positive development	*Cortinarius orellanus*
[45]	October 1994	Italy	1	M/53	NC/H 18	Oliguria	Activated charcoal, intravenous fluids, plasmapheresis, hemodialysis	Serum creatinine: 97.5 µmol/L at H 30; Percutaneous renal biopsy at D 8 reveal acute tubular necrosis with interstitial oedema	-	Renal allograft at Mo 17	*Cortinarius orellanus*
[46]	August 1997	Ireland	2	F/66	D5/D10	Vomiting, colicky, diarrhea, abdominal pain, oliguria, hyponatremia, proteinuria	Hemodialysis, prednisolone, intravenous *N*-acetylcysteine	Past of left sided hydronephrosis; Serum creatinine: 1032 µmol/L at D 10; Uremia: 32.8 mmol/L at D 10	-	Renal function normal	*Cortinarius orellanus*
				F/38	NC/NC	NC	NC	Serum creatinine: 376 µmol/L	-	NC	
[29]	NC	Australia	3	M/17	1–2 weeks/2–3 week	Nausea, diarrhea, anuria	Hemodialysis, methylprednisolone, prednisolone	Past of drug addict; Voluntary ingestion looking for hallucinogenic effects; Serum creatinine: 1970 µmol/L; Uremia: 44.3 mmol/L; Renal biopsy reveal acute interstitial nephritis	-	Death of pulmonary oedema at Mo 5	NC
				M/26	D 2/D 4	Vomiting, epigastric, back and bilateral loin pain, acute renal failure, dehydration, oliguria	Intravenous fluids, intravenous frusemide, hemodialysis	Past of polysubstance abuse; Voluntary ingestion of 12 uncooked mushrooms looking for hallucinogenic effects; Serum creatinine: 1050 at D 4; Uremia: 19.5 mmol/L at D 4; Renal biopsy at D 20 reveal edematous interstitial fibrosis	-	Peritoneal dialysis for 15 months	
				M/16	D 4/D 8	Vomiting, acute renal failure, oliguria, dehydration	Intravenous fluids	Serum creatinine: 760 at D8; Uremia: 15.6 mmol/L at D 8	-	Positive development; Patient failed to attend a scheduled outpatient appointment	
[27]	December 1985	Australia, Tasmania	2	M/NC	NC/D 7	Kidney failure	Dialysis	-	-	Kidney transplantation	*Cortinarius eartoxicus*
					NC/NC	NC	NC	-	-	Renal function normal	
[7]	NC	Germany	2	M/30	D 4/D 6	Nausea, vomiting, back pain, proteinuria	Intravenous *N*-acetylcysteine, selenium, hemodialysis	Consumption of remaining mushroom 3 days after the first; Serum creatinine: 459.7 µmol/L at D6, 928 µmol/L at D 7; Uremia: 12.9 mmol/L at D 6, 21.1 mmol/L at D 7	-	Renal function normal	*Cortinarius speciosissimus*
				F/29	NC/D 6	Nausea, back pain, proteinuria	Intravenous *N*-acetylcysteine, selenium	Consumption of remaining mushroom 3 days after the first; Serum creatinine: 88.4 µmol/L at D 6; Uremia: 5.4 mmol/L at D 6	-	Renal function normal	
[23]	NC	United States, Michigan	1	F/53	D 3/D 9	Vomiting, diarrhea, oliguria	Intravenous sodium bicarbonate, sodium polystyrene sulfonate, hemodialysis	Consumption of 6 mushrooms; Serum creatinine: 1220 µmol/L at D 9; Uremia: 14.6 mmol/L at D 9; Renal biopsy at D 14 reveal acute tubular necrosis	-	Peritoneal dialysis 5 time a week	*Cortinarius orellanosus*
[20]	NC	Norway	8	4 M–4F/between 44 and 74	D 2/D 7	Gastrointestinal disorder, headache, myalgia, acute renal insufficiency, oliguria	5 dialysis; 6 steroids + *N*-acetylcysteine	Serum creatinine: 150–1627 µmol/L	-	3 chronic hemodialysis; 5 partial recovery	*Cortinarius orellanus*
[47]	NC	Austria	2	F/62	D 2/D 6	Nausea, vomiting, epigastric pain acute renal failure, anemia	Prednisolone, intravenous *N*-acetylcysteine	Serum creatinine: 587 µmol/L at D 6; Uremia: 28.2 mmol/L at D 6; Renal biopsy at D 8 reveal acute interstitial nephritis	TLC on biopsy specimen failed to detect orellanin	Prednisolone for 103 D; Renal function normal	*Cortinarius speciosissimus*
				M	D 2/D 6	Nausea		Serum creatinine: 890 µmol/L at D 6; Uremia: 36.8 mmol/L at D 6	-		
[48]	NC	Wales	1	M/43	D 4/D 14	Nausea, vomiting, diarrhea, myalgia, fever, anuria, dehydration, hematuria, leukocyturia, acute kidney injury	Hemodialysis, methylprednisolone, prednisolone	Blood creatinine: 2650 µmol/L at D 14; Uremia: 50 mmol/L at D 14; Kidney biopsy reveal severe interstitial nephritis at D 17	-	Kidney transplantation at Mo 20	*Cortinarius speciosissimus*

N: number of patients; NC: not communicated; NA: not applicable; F: female; M: male; D: day; W: week; Mo: month; Y: year.

**Table 2:** Analytical methods for orellanine detection.

Ref.	Matrix	Separation	Detection	Qualitative/Quantitative	LOD	LOQ	Linearity	Extraction Recovery	Additional Analytical Information
[14]	Mushrooms	TLC	UV	Qualitative	NA	NA	NA	NA	-
[49]	Mushrooms	TLC	UV (254 nm)	Qualitative	NA	NA	NA	NA	-
[50]	Mushrooms, mouse serum and kidney	HPLC	Electrochemistry (Working electrode: glassy carbon TL-5A; Reference electrode: Ag/AgCl; Working potential: 900 mV)	Quantitative	500 pg	NC	50–500 ng on column	Alleged to 100% on overloaded mouse serum and directly injected, 25% for mouse kidney	Column: (200 mm × 4.6) 5 µm Nucleosil C18; Flow rate: 2 mL/min; Mobile phase: 0.05 citrate-phosphate buffer pH 4.5, 15.4% MeOH and PIC B6 1-hexane sulphonic acid 5 mM
[21]	Mushrooms	TLC	Spectrofluorometry (λ_excitation_ = 396 nm; λ_emission_ = 447 nm)	Quantitative	NC	NC	NC	NC	-
		HPLC	MS	Qualitative	NA	NA	NA	NA	
		-	NMR	Qualitative	NA	NA	NA	NA	
[22]	Mushrooms	-	Polarography (Working electrode: dropping mercury; Reference electrode: saturated calomel)	Qualitative	NA	NA	NA	NA	-
[51]	Mushrooms	HPLC	UV (260, 290 nm)	Quantitative	40–50 pg on column	NC	5–500 ng on column	NC	Columns: (150 mm × 4.6) 5 µM Rosil CN and (150 mm × 3.9) 5 µM µBondapak C18; Flow rate: 0.5 mL/min and 0.8 mL/min; Mobile phase: H_3_PO_4_ pH 1 and H_3_PO_4_ pH/ACN (94/6 *v*/*v*); 1-octane-sulphonic acid 2.5 Mm; RT: 4.43 min and 6.58
[24]	Biological fluids and renal biopsy	TLC	Spectrofluorometry in 2D (λ_excitation_ = 399 nm; λ_emission_ = 447 nm)	Quantitative	10 ng	NC	NC	NC	-
[28]	Mushrooms	TLC	Spectrofluorometry (λ_excitation_ = 400 nm; λ_emission_ = 450 nm)	Quantitative	15 ng deposit	NC	NC	NC	-
		Electrophoresis	Spectrofluorometry (λ_excitation_ = 400 nm; λ_emission_ = 450 nm)	Quantitative	25 ng deposit	NC	NC	NC	
		-	ESR	Quantitative	5000 ng	NC	NC	NC	
[41]	Urine, blood and renal biopsy	TLC	UV (366 nm)	Semi quantitative	≈ 10 ng	NC	NC	NC	-
[52]	Mushrooms	TLC	UV (365 nm)	Semi quantitative	≈ 50 ng deposit	NC	NC	NC	-
		HPLC	Photodiode (288 nm)	Quantitative	NC	NC	NC	NC	Preparative column: (115 mm × 13 mm) C18; Flow rate: 1 mL/min; Mobile phase: ACN/H_2_O (5/95 *v*/*v*) pH 1 1% TFA; RT: 6.5 min
		HPLC	ESI-MS	Quantitative	NC	NC	NC	NC	Flow rate: 10 µL/min direct MS source
[10]	Mushrooms and rat plasma	HPLC	ESI-MS/MS (triple Q) (253 to 191; 253 to 219; 253 to 163 *m*/*z*)	Quantitative	4.9 µg/L	NC	4.9–5000 µg/L	≈ 91% mushrooms ≈ 60% plasma	Column: (50 mm × 2.1 mm) 1.8 µm Eclipse Plus C18 RRHD; Flow rate: 0.2 mL/min; Mobile phase: 4 mM ammonium formate pH 2.5 (A), MeOH 0.2% HCOOH (B)
			ESI-MS/MS (QTOF)	Quantitative	4.9 µg/L	NC	4.9–5000 µg/L		Flow rate: 0.2 mL/min; Mobile phase: 5 mM ammonium formate/MeOH (90/10; *v*/*v*) 0.02% HCOOH (A), 5 mM ammonium formate in MeOH 0.02% HCOOH (B)
[53]	Rat gastric content	HPLC	(−) ESI-MS/MS (triple Q) (Scan range: 120–600 *m*/*z*)	Quantitative	NC	NC	NC	NC	Column: (50 mm × 2.1 mm) 2 µm Ascentis Express C18; Flow rate: 0.25 mL/min; Mobile phase: H_2_O 0.1 N HCOOH (A), ACN (B)
[54]	Rat gastric content	GC	MS with Supersonic Molecular Beam	Qualitative	NC	NA	NA	NA	Column: (4 m × 0.25 mm ID), 0.1 µm VF-5HT; Flow rate: 8 mL/min; T injector: 200 °C; GC oven: 120–300 °C at 30 °C/min
[26]	Mushrooms	HPLC	UV–visible (295 nm)	Quantitative	17000 ng/g	NC	17000–680000 ng/g	78.3%	Column: (150 mm × 4.6 mm) 3 µm PLRP-S C18; Flow rate: 0.3 mL/min; Mobile phase: 4 mM ammonium acetate (A), MeOH (B)
			ESI-MS/MS (triple Q) (253 to 163; 253 to 191; 253 to 219; 253 to 236 *m*/*z*)	Quantitative	30 ng/g	NC	6800–13600 ng/g	85.0%	Column: (250 mm × 4.1 mm) 10 µm Hamilton PRP-1; Flow rate: 0.4 mL/min; Mobile phase: H_2_O 1% HCOOH (A), ACN (B)
[55]	Mice kidney	HPLC	UV–visible	Quantitative	NC	10 µg/g of tissue	15–50 µg/g of tissue	NC	-
		HPLC	ESI-MS/MS (triple Q) (235 to 236 *m*/*z*)	Quantitative	20 ng/g	NC	NC	91%	
[56]	Standard solution	-	PSI-HR-MS/MS (253.0468 to 219.0404 *m*/*z*)	Qualitative	NA	NA	NA	NA	-

NA: not applicable; LOD: limit of detection; LOQ: limit of quantification; NC: not communicated.

**Table 3:** Cases of amatoxines poisoning.

Ref.	Date of Intoxication	Country	*N*	Sex/Age	Offset of Symptoms/Delay before Hospitalization	Symptoms	Treatment	Notes	Toxin Quantification	Outcome	Mushroom Specie
[80]	9 October 1944	Great Britain	4	F/26	H 6/H 18	Vomiting, diarrhea, coma	Gastric lavage, glucose, atropine, insulin	Uremia: 25 mmol/L at D 3	-	Death at H 111 of gastric hemorrhages, kidney and liver failure	*Amanita phalloides*
	9 October 1944			F/38	H 6/H 18	Vomiting, diarrhea, cyanosis	Gastric lavage, atropine, magnesium sulfate, insulin, glucose, nikethamide, percortone	Uremia: 23.3 mmol/L at D 3	-	Death at H 76 of gastric hemorrhages, kidney and liver failure	
	25 September 1944			F/57	H 8/D 1	Vomiting, diarrhea, abdominal pain, coma	Castor oil, intravenous plasma	-	-	Death at H 126 of kidney and liver failure	
	18 August 1945			F/6	NC/D 3	Vomiting, diarrhea, cyanosis	Gastric lavage, atropine	-	-	Death at H 60 of kidney and liver failure	
[81]	1943	Great Britain	3	F/≈ 25	NC	Jaundice, hallucinations	NC	-	-	Positive development	*Amanita phalloides*
				F/NC	NC/D 2	Vomiting, diarrhea, abdominal pain, severe muscular cramps, constipation, anorexia	NC	-	-	Positive development	
				F/5	NC/D 2	Vomiting, diarrhea, delirium, coma	NC	-	-	Death at D 2 of liver degeneration	
[82]	September 1961	United States, Washington DC	1	M/8	NC	Vomiting, lethargy, inability to see, irrational response, cerebral oedema	NC	Visit to the hospital because of head trauma after a bike fall	Amatoxins identification in the liver by TLC	Death on the hospital D 4	NC
[83]	13 November 1962	United States, California	2	M/43	H 5/NC	Nausea, vomiting, diarrhea, oliguria, renal failure, dehydration, distention of the abdomen, hyperventilation, disorientation, hallucinations, coma, cyanosis, apnea	Peritoneal dialysis, intravenous plasma, antibiotics	Past of alcoholism; Serum creatinine: 1202 µmol/L at D 3; Uremia: 33 mmol/L at D 3; Septicemia complication	-	Death at D 12 of kidney and liver failure, central nervous system complication	Possible *Amanita phalloides*
	4 October 1962			F/43	H 2/D 4	Vomiting, slight lacrimation, acute renal failure, anuria, pruritus, dyspnea, confusion, hyponatremia, pulmonary oedema,	Atropine, peritoneal dialysis	Uremia: 10 mmol/L at D 4; Renal biopsy at D 43 reveal renal tubular necrosis	-	Positive development	
[84]	NC	United States, California	5	M/77	H 6/D 1	Vomiting, diarrhea, abdominal pain, severe cramping, hypotension, rapid supraventricular tachycardia, anuria, muscular hyperactivity, coma, hypoglycemia	Atropine intramuscularly, intravenous fluids, digitalis, sodium bicarbonate, dextrose, *Amanita phalloides* antitoxin, peritoneal dialysis	-	-	Death at D7 of kidney and liver failure	*Amanita verna*
				1 M and 3 F/20, 60, 62, 63	H 10–15/NC	Gastrointestinal disorders, oliguria, dehydration, neutropenia	NC	-	-	Positive development	*Amanita verna, Amanita phalloides*
[70]	Between 1968 and 1974	United States, California	28	NC/Between 14 months and 87 years old	NC	Nausea, vomiting, diarrhea, abdominal pain	Supportive care, 14 thioctic acid	-	Amatoxins identification in mushrooms by TLC	8 deaths; 20 Positive development	*A. phalloides, A. virosa, A. verna* et *G. autumnalis*
[66]	NC	Switzerland	8	4 H–4 F/between 16 and 55	NC/H 16	Severe gastrointestinal disorders	Dialysis, hemoperfusion, penicillin, vitamin C	ALT peak at 1920 IU/L at D 3 for one patient	-	Positive development	*Amanita phalloides*
[85]	Fall 1981	United States, California	10	M/45	H 8/H 12	Nausea, abdominal cramping, diarrhea, dehydration, oliguria, encephalopathy, respiratory arrest, seizures, hepatic coma	Rehydration, vitamin K, thioctic acid, diazepam, phenytoin	Consumption of 2 or 3 mushrooms; AST at D 6: 4220 U/L; ALT at D 6: 7272 U/L; Serum creatinine at D 11: 336 µmol/L	-	Death at D 12 of kidney and liver failure, cerebral oedema	NC
				M/80	D 1/H 48	Nausea, vomiting, diarrhea, dehydration, confusion, hypotension, supraventricular tachycardia, oliguria, encephalopathy, coma	Rehydration, dextrose	Serum creatinine at D 2: 380 µmol/L; Uremia: 8.7 mmol/L; AST at D 4: 2410 U/L; ALT at D 4: 2500 U/L; Septicemia developed on D 7	Amatoxins identification positive on the meal	Death at D 9	
				M/39	H 12/D 4	Vomiting, diarrhea, dehydration, hematemesis, cardiopulmonary arrest	Rehydration	AST at D 4: 4860 U/L; AST at D 5: 2820 U/L; ALT at D 5: 3220 U/L; Serum creatinine at D 5: 513 µmol/L	-	Death at D 6 of multiorgan failure	
				M/18	H 8–10/NC	Nausea, vomiting, abdominal cramps, diarrhea, dehydration, bradycardia, hypotension	Rehydration, dextrose, dexamethasone, vitamin K, temporary transvenous pacemaker	Consumption of 10 mushrooms; AST at D 3: 5280 U/L; ALT at D 3: 5100 U/L	-	Positive development	*Amanita phalloides*
				3 M–3 F/21–37	H 8–12/NC	Nausea, vomiting, abdominal cramps, diarrhea	Supportive care, activated charcoal	Laotian refugees; AST peak between 617 and 2565 U/L; ALT peak between 648 and 5870 U/L	-	Positive development	*Amanita* species
[86]	November 1981	Italy	1	F/21	H 10/NC	Nausea, vomiting, abdominal pain, diarrhea	Plasmapheresis, forced diuresis	8 months of pregnancy	α-amanitin = 18.5 ng/mL in the serum by HPLC; No amatoxins in amniotic fluid	Positive development	*Amanita phalloides*
[87]	28 February 1983	United States, California	1	F/3	H 8/D 2	Nausea, vomiting, abdominal pain, diarrhea, hypotension, oliguria, hematuria, encephalopathy grade III, coma	Rehydration, charcoal slurry, lactulose, dopamine and dobutamine hydrochloride, antibiotics, methylprednisolone, charcoal hemoperfusion	Consumption of 2 tablespoons of mushrooms; AST at D 2: 16,648 U/L; ALT at D 2: 9844 U/L; Left hepatic lobectomy on the transplant liver because of necrosis at D 9	-	Orthotopic liver transplantation at D 5 + neurological deficits	*Amanita ocreata*
[88]	1982–1983	United States, California	21	10 M–11 F/5–82	H 6–29/D 1–12	Nausea, vomiting, abdominal cramps, diarrhea	Supportive care, activated charcoal, 5 dexamethasone	AST peak: 77–11674 U/L; ALT peak: 72–9233 U/L	Amatoxins identification positive in serum of 3 patients by RIA	2 deaths; 19 Positive development	*A. phalloides, A. ocreata,* L. *clypeolaria*
[67]	NC	United States, California	2	F/19	H 9/NC	Nausea, vomiting, diarrhea, abdominal pain, hepatic encephalopathy	Rehydration, gastric lavage, charcoal, dialysis	Consumption of 6 ounces of mushrooms; AST: 1608 U/L; ALT: 2600 U/L	-	Orthotopic liver transplantation	*Amanita phalloides*
				M/45	H 7/NC	Nausea, vomiting, diarrhea, oliguria, encephalopathy grade III	Rehydration, gastric lavage, charcoal, hemodialysis	Consumption of ≈ 250 g of mushrooms; AST: 3800 U/L; ALT: 5600 U/L	-	Orthotopic liver transplantation	
[89]	22 October 1988	United States, Oregon	5	2 M–3 F/33–52	H 7–11/<H 24	Nausea, vomiting, diarrhea, abdominal cramps, dehydration, hypophosphatemia, 2 encephalopathy grade I and 2 encephalopathy grade II	Rehydration, silymarin, penicillin	Consumption of 60–100 mushrooms; 1 diabetic had undergone previous cholecystectomy and pelvic surgery; 1 pulmonary tuberculosis	-	4 liver transplantation at D 5–7; 1 death	*Amanita phalloides*
[90]	1984–1989	France	45	22 M–23 F/2–81	H 6–24/NC	Gastrointestinal disorders; 43 hepatic injury; 6 functional renal failure	Supportive care, penicillin G, silibinin; 1 hemodialysis; 2 gastric lavage	AST peak: 380–17000 U/L; ALT peak: 520–16,000 U/L	Amatoxins identification in biological matrix by HPLC-UV	2 liver transplantation at D 5; 8 deaths; 35 positive development	*Amanita phalloides*
[91]	NC	United States, New York	4	F/90	H 12/H 30	Nausea, vomiting, diarrhea, weakness, hypotension, hepatic failure	Rehydration, penicillin, cimetidine, activated charcoal, vitamin K	Past of hypertension, permanent pacemaker; Serum creatinine at D 2: 124 µmol/L; Uremia at d2: 16.1 mmol/L; AST at D 7: 4099 U/L; ALT at D 7: 5394 U/L	Amatoxins identification positive in admission and post-mortem serum	Death at D 7 of hepatic failure	*Amanita/Lepiota* species
				M/64	H 12/H 30	Nausea, vomiting, abdominal cramps	Rehydration, penicillin, cimetidine, activated charcoal, vitamin K	Serum creatinine at D 2: 159 µmol/L; Uremia at D2: 11.8 mmol/L; AST at D 5: 5620 U/L; ALT at D 5: 8620 U/L	-	Hepatitis	
				F/40 M/42	H 3/H 18	Nausea, vomiting, diarrhea	rehydration, prochlorperazine, charcoal, penicillin, charcoal hemoperfusion, heparin	Consumption of 4–6 mushrooms	-	Positive development	*Lepiota chlorophyllum*
[68]	1991–1992	France	4	F/27	H 10/NC	Nausea, vomiting, abdominal pain, diarrhea, encephalopathy grade I, anemia, leukopenia	rehydration, silibinin, ceftazidime, hemodialysis	Consumption of 300 g of mushrooms; AST at D2: 2990 U/L; ALT at D2: 2730 U/L	-	Liver transplantation, chronic renal failure, myocardiopathy	*Lepiota helveola*
				M/35	H 12/NC	Vomiting, diarrhea, abdominal pain, hepatitis	NC	Consumption of alcohol during the meal	-	Positive development	*Lepiota brunneolilacea*
				F/33	H 12/NC	Vomiting, diarrhea, abdominal pain, dehydration, hepatic cytolysis, disorientation, asterixis	NC	AST at D 2: 5800 U/L; ALT at D 2: 2700 U/L	-	Liver transplantation at D 4	
				F/8	H 12/NC	Vomiting, diarrhea, abdominal pain, dehydration, encephalopathy grade III	rehydration, albumin	AST at D 2: 1416 U/L; ALT at D 2: 1560 U/L; ALT at D 3: 5082 U/L	-	Orthotopic liver transplantation at D 5	
[92]		Turkey	3	3 M/9, 11, 14	H 12/H 30	Nausea, vomiting, abdominal pain, diarrhea, dehydration	Gastric lavage, charcoal hemoperfusion, rehydration, lactulose, penicillin, streptomycin, forced diuresis, dexamethasone, vitamins, hemodialysis	Consumptions of ≈ 80 g of mushrooms; AST peak: 276–1760 U/L; ALT peak: 388–3450 U/L	α-amanitin identification positive in serum by TLC	Positive development	*Amanita phalloides*
[93]	27 December 1996 to 5 January 1997	United States, California	10	9 M–1 F/ 12/68	H 8–26/D 2–8	Nausea, vomiting, diarrhea, abdominal cramps, weakness,	rehydration, H_2_-blockers, activated charcoal, penicillin, *N*-acetylcysteine, vitamin K, hemodialysis	AST peak 594–6998 U/L; ALT peak: 930–7120 U/L	-	2 deaths at D 7 and D 9 of multiorgan failure	*Amanita phalloides*
[94]	1995	Australia	2	M/46	NC/D 1	Vomiting, diarrhea, hepatic and renal failure	rehydration, penicillin, *N*-acetylcysteine	Consumption of 8 mushrooms; ALT at D 3: >10,000 U/L; Serum creatinine at D 3: 535 µmol/L	-	Death at D 6 of hepatic failure waiting for a liver transplantation	*Amanita phalloides*
	1998			M/39	H 18/H 36	Nausea, vomiting, diarrhea, dehydration,	rehydration, penicillin, *N*-acetylcysteine	Consumption of 3 mushrooms; ALT peak at D 3: 8199 U/L; Serum creatinine at D 2: 102 µmol/L	-	Positive development	
	1988–1997		5	3 M–2 F/7–45	D 1–2/NC	Vomiting, diarrhea	rehydration, activated charcoal, penicillin	1 patient ALT peak: 2938 U/L	-	Positive development	
[95]	NC	Thailand	5	F/36	H 12/NC	Nausea, vomiting, diarrhea, jaundice, acute liver failure, hepatic encephalopathy	Supportive care, vitamin K, neomycin, lactulose	Serum creatinine: 132.6 µmol/L; Uremia: 2.2 mmol/L; AST: 3400 U/L; ALT: 3930 U/L	-	Death at D 6	*Amanita virosa*
				M/8	H 12/NC	Nausea, vomiting, diarrhea, jaundice, hepatic encephalopathy, convulsions, gastrointestinal bleeding, hypoglycemia	rehydration	Serum creatinine at D 4: 35.4 µmol/L; Uremia at D 4: 0.8 mmol/L; ALT at D 4: 1738 U/L	-	Death at D 5	
				M/36	NC	Nausea, vomiting, diarrhea, acute liver failure, hepatic encephalopathy	NC	-	-	Death at D 4–6	
				M/11							
				F/6							
[62]	NC	United States, Ohio	4	F/53	H 10/NC	Nausea, vomiting, abdominal cramps, diarrhea, hypokalemia, anemia, hepatic encephalopathy grade III	Charcoal hemoperfusion, penicillin G, thioctic acid, vitamin C, dexamethasone, Pepcid	Consumption of ≈ 900 g of mushrooms; Past of breast cancer, left mastectomy; AST peak: 1494 U/L; ALT peak: 1277 U/L	-	Orthotopic liver transplantation at D 4 + mild renal insufficiency	*Amanita virosa*
				M/25	NC/H11	Vomiting, abdominal cramps, diarrhea	Charcoal hemoperfusion, forced diuresis, hydration, vitamin K, decadron, penicillin G, vitamin C, cimetidine	Consumption of 40–50 g of mushrooms	-	Positive development	
				M/35	H 10½/NC	Nausea, vomiting, diarrhea, abdominal pain	Charcoal hemoperfusion, fluid and electrolyte repletion, penicillin G, dexamethasone	Consumption of 40–50 g of mushrooms; AST peak: 761 U/L; ALT peak: 531 U/L	-	Positive development	
				M/47		Nausea, vomiting, diarrhea, abdominal pain	Charcoal hemoperfusion rehydration, electrolyte repletion, penicillin G, dexamethasone, vitamins	AST peak: 154 U/L; ALT peak: 122 U/L	-	Positive development	
[96]	NC	Japan	1	M/6	H 6–10/H 36	Nausea, vomiting, diarrhea, abdominal pain, dehydration, hepatic insufficiency, mild proteinuria, glycosuria, hematuria	rehydration, plasma exchange, hemodiafiltration, activated charcoal	AST peak at H62: 18450 U/L; ALT peak at H62: 13,554 U/L	Amatoxins identification negative in urine and blood at H80; Amatoxins identification positive in mushrooms by HPLC	Positive development	Possible *Galerina fasciculata*
[97]	NC	France	1	F/22	H 2/H 13	Nausea, vomiting, diarrhea, abdominal pain	rehydration, silymarin, activated charcoal, *N*-acetylcysteine, vitamins, antibiotics, fungizone	2 months of pregnancy; AST peak at H53: 3200 U/L; ALT peak at H67: 4127 U/L	-	Positive development	*Amanita phalloides*
[98]	NC	Switzerland	1	F/61	H 12–16/H 36	Nausea, vomiting, diarrhea, dehydration, hypoglycemia,	rehydration, vitamin K, penicillin G, silibinin, *N*-acetylcysteine	Dried and frozen mushrooms during 7–8 months; Serum creatinine at H 48: 270 µmol/L; AST at H 48: 1424 U/L; ALT at H 48: 2326 U/L	Amatoxins identification positive in urine at D 4: 37.3 µg/L	Death at D4 of liver and renal failure (patient declined the liver transplantation)	*Amanita phalloides*
[99]	NC	Turkey	2	M/44	H 8/NC	Nausea, diarrhea, abdominal pain, encephalopathy grade III, hepatitis	NC	Transplanted liver necrosis; AST at D 10 postoperative: 10,270 U/L; ALT at D 10 postoperative: 5670 U/L	-	Death at D 10 after an orthotopic liver transplantation	*Amanita phalloides*
				F/20	NC/D 2	Nausea, vomiting, diarrhea, confusion, lethargy, agitation, hepatic encephalopathy grade II, hepatitis	NC	-	-	Orthotopic liver transplantation	
[100]	NC	Germany	1	F/64	NC	Hepatic encephalopathy grade III	NC	Obesity, hypertension, chronic heart failure	-	Hepatocyte transplantation	*Amanita phalloides*
[101]	NC	Turkey	1	M/11	H 24/NC	Nausea, vomiting, abdominal cramps, diarrhea, metabolic acidosis, fever, jaundice, unconsciousness, hypotonia, hepatic encephalopathy grade III	Gastric lavage, activated charcoal, vitamin K, penicillin G, bicarbonate, ampicillin, lactulose, vitamin C, plasmapheresis	AST peak: 774 U/L; ALT peak: 200 U/L	-	Orthotopic liver transplantation	*Amanita phalloides*
[102]	NC	France	5	M/NC	H 9/NC	Vomiting, diarrhea, abdominal pain, dehydration	Penicillin G, silimarin	AST at H 48: 150 U/L; ALT at H48: 270 U/L	Amatoxins identification positive by RIA in urine at H 24: 5.99 µg/L	Positive development	*Amanita phalloides*
				F/NC	H 11/NC	Vomiting, diarrhea	NC	-	Amatoxins identification positive in urine at H 27: 14.3 µg/L; Negative in serum by RIA		
				M/NC	H 14/NC				Amatoxins identification positive in urine at H 27: 11.6 µg/L; Negative in serum by RIA		
				M/NC	D 1/D 1	Diarrhea, liver and renal insufficiency	*N*-acetylcysteine	AST at H 60: 1014 U/L; ALT at H 60: 2645 U/L	Amatoxins identification negative in serum, urine and feces at H72 < 1.5 µg/L		
			3	NC	NC	NC	NC	NC	Amatoxins identification in urine at H > 36; 1.5 < X < 5 µg/L	NC	NC
[103]	1988–2002	Italy	111	57 M–54 F/18–94	H ≈ 12/H 30–45	Nausea, vomiting, diarrhea	rehydration, glucose, electrolyte repletion, vitamin K, activated charcoal, dexamethasone, penicillin G	AST peak: 4330 U/L; ALT peak: 5428 U/L	Amatoxins identification positive in urine in 62 patients	2 deaths at D 11 and D 29	Amatoxins-containing species
[104]	2000–2004	Czech Republic	34	17 M–17 F/1–73	H 1–24/H 1–168	Vomiting, diarrhea, abdominal cramps, weakness, hepatic failure, coagulopathy, encephalopathy, renal failure	Gastric lavage, activated charcoal, penicillin G, thioctic acid, hemoperfusion, hemodialysis, *N*-acetylcysteine, silymarin, forced diuresis	5 intentional ingestion (suicide); 5 alcohol abuse	-	3 deaths at D 5 of cardiac arrest, D 5 during liver transplantation and M 19 of renal damage; 14 persistent hepatic or renal damage	*Amanita phalloides*
[105]	NC	Turkey	1	F/16	H 7/D 3	Nausea, vomiting, abdominal pain, diarrhea, lethargy, liver failure	Supportive care, silibinin, oral charcoal, plasmapheresis	-	-	Liver transplantation at D 7	*Amanita phalloides*
[106]	NC	Tunisia	4	F/6	H 7/NC	Vomiting, diarrhea, abdominal pain,	-	-	-	Death at D 1 before arriving at emergencies of liver failure	*Lepiota brunneoincarnata*
				M/15	NC/H 7	Vomiting, diarrhea, fever, hypovolemia, hepatic cytolysis, hematemesis,	rehydration	AST peak at D 3: 5400 U/L; ALT peak at D 3: 5500 U/L	-	Death at D 3 of liver failure with brain oedema	
				F/12	NC/H 12	Vomiting, diarrhea, abdominal pain, coma, brain oedema, hepatic cytolysis	NC	AST peak at D 3 > 10000 U/L; ALT peak at D 3 > 10,000 U/L	-	Brain death at D 3; Death at D 11 of multiorgan failure	
				M/3	H 7/NC	Vomiting, diarrhea, abdominal pain, hepatic cytolysis, acute renal failure, metabolic acidosis	rehydration, vitamin K	AST peak at D 3 > 10,000 U/L; ALT peak at D 3 > 10,000 U/L	-	Death at D 4 of multiorgan failure	
[72]	January 2000 to October 2010	Germany	79	NC	Medial H 14.5/Medial H 29.4	Nausea, vomiting, diarrhea, abdominal pain, coagulopathy	9 activated charcoal, laxative, 10 silibinin, 3 penicillin, 6 *N*-acetylcysteine	AST medial peak: 3242 U/L; ALT medial peak: 3907 U/L	10 amatoxins identification positive in urine by ELISA: 15.3–125 µg/L (4 after H 48)	10 positive development	NC
[107]	March 1992 to November 2009	Portugal	10	4 M–6 F/16–75	H 7–12/<H 48	Vomiting, diarrhea, abdominal pain, encephalopathy grade I, acute liver failure	Supportive care, silibinin, penicillin G, *N*-acetylcysteine, hemodialysis, hemodiafiltration	AST medial peak: 5295 U/L; ALT medial peak: 6919 U/L	-	4 deaths (3 liver transplantation); 3 liver transplantation alive; 3 positive development	*Amanita phalloides*
[108,109]	January 1995 to December 2009	Switzerland	32	20 M–12 F/1, 4–74	H 1, 25–6/NC	Nausea, vomiting, diarrhea, dehydration, acute liver failure, encephalopathy grade I	Activated charcoal, silibinin, gastric lavage, forced diuresis, laxatives, penicillin G, *N*-acetylcysteine	2 intentional ingestions	Amatoxins identification positive in urines by ELISA; 1.6 < X < 118 µg/L	5 deaths at D 3–9 of liver failure; 27 positive development	*Amanita phalloides, Amanita virosa*
[110]	NC	Turkey	1	M/63	H 7–8/H 36	Nausea, vomiting, diarrhea, weakness, dehydration	Gastric lavage, activated charcoal, hemodialysis, rehydration, silibinin, *N*-acetylcysteine, penicillin G, multivitamin	Chemotherapy + surgery for a colon carcinoma 2 months before; Liver transplantation refused because of colon carcinoma; AST peak at H 90: 3570 U/L; ALT peak at H 90: 3282 U/L	-	Death at H 134 of cardiac arrest	*Amanita phalloides*
[77]	NC	United States, Massachusetts	2	F/72	H 28 (after the 1st meal)/D 2	Vomiting, diarrhea, abdominal pain	Activated charcoal, *N*-acetylcysteine, penicillin G, silibinin, cimetidine	Past of hypertension; Consumption of the same mushroom on 2 consecutive days; AST peak at H 64: 9640 U/L; ALT peak at H 64: 9360 U/L	-	positive development	*Amanita ocreata*
				M/45	H 14/ D 1			Past of hypertension; AST peak at H 60: 2868 U/L; ALT peak at H 60: 4212U/L	-		
[8]	NC	Australia	1	F/58	H 9/D 1	Vomiting, diarrhea, coagulopathy liver failure, encephalopathy	Silibinin, penicillin G, *N*-acetylcysteine	Consumption of 6 mushrooms; AST peak at H 96: 1842 U/L; ALT peak at H 96: 2143 U/L	-	Death at D 5 of fulminant liver failure	*Amanita phalloides*
[111]	November 2011	France	3	M/8	NC/H 9	Vomiting, diarrhea, abdominal cramps, asthenia, fever, confusion, dehydration,	Activated charcoal, penicillin G, silibinin, *N*-acetylcysteine	AST at D 4: 1018 UI/L; ALT at D 4: 3205 UI/L	-	positive development	*Lepiota brunneoincarnata*
				F/11	NC/H 9	Vomiting, abdominal cramps		-			
[112]	January 2002 to December 2012	Italy	242	NC/Medial 53	NC	Gastrointestinal disorders	N-acetylcysteine, forced diuresis, activated charcoal	α-amanitin identification positive in urine: medial: 39.21 µg/L		5 Deaths; 5 Liver transplantation; 232 positive development	Amatoxins-containing species
[76]	NC	United States, New York	1	M/65	H 14/NC	Vomiting, diarrhea,	rehydration, antiemetics, *N*-acetylcysteine, silimarin, biliary drainage, octreotide	AST peak: 5102 U/L; ALT peak: 2546 U/L	-	positive development	*Amanita bisporigera*
[75]	NC	Republic of Macedonia	8	M/54	H 24/NC	Nausea, vomiting, diarrhea, weakness, fatigue, confusion, neurological reaction depression, liver encephalopathy grade III, renal failure	Activated charcoal, *N*-acetylcysteine, vitamins, penicillin G, H_2_ blocker, ornicetil, hemoperfusion, plasma exchange, plasmapheresis	Consumption of the same mushroom on 2 occasions; AST peak: 4714 U/L; LT peak: 5824 U/L; Serum creatinine peak: 180,000 µmol/L; Uremia: 13.3 mmol/L	-	Death at hospitalization D 5 of hepatorenal failure	
				M/30	NC/NC	Nausea, vomiting, diarrhea, weakness, fatigue, confusion, neurological reaction depression, liver encephalopathy grade III, renal failure		Consumption of the same mushroom on 2 occasions; AST peak: 3600 U/L; ALT peak: 6025 U/L; Serum creatinine peak: 230000 µmol/L; Uremia: 1.9 mmol/L		Death at hospitalization D 5 of hepatorenal failure	*Amanita verna*
				F/75	H 10/NC	Nausea, vomiting, diarrhea, weakness, fatigue, abdominal pain		AST peak: 307 U/L; ALT peak: 321 U/L		positive development	
				F/54	NC/D 1	Nausea, vomiting, diarrhea, abdominal pain		Consumption of ≈ 300 g of mushrooms			
				F/31	NC/D 1	Nausea, vomiting, diarrhea, weakness, fatigue		Consumption of ≈ 300 g of mushrooms; Cholecystectomy in the past; AST peak: 306 U/L; ALT peak: 293 U/L			
				M/34	H 10/NC	Nausea, vomiting, diarrhea, weakness, fatigue		Consumption of ≈ 300 g of mushrooms			
				M/23 F/32	NC/NC	Nausea, abdominal pain	Activated charcoal, *N*-acetylcysteine, vitamins, penicillin G, H_2_ blocker, hemoperfusion,	-			
[113]	August 2014	Sweden	6	NC	NC	Nausea, vomiting, diarrhea, liver impairment	Silibinin, *N*-acetylcysteine	Syrians refugee	Amatoxins identification positive in urine	positive development	*Amanita virosa*
[114]	NC	Turkey	1	M/61	H 8–9/H 24	Nausea, vomiting, diarrhea, abdominal pain, fatigue, dehydration	rehydration activated charcoal, penicillin G	Voluntary ingestion of 2 caps in order to test the toxicity ≈ 21.3 mg amatoxins AST peak at H 72: 1777 U/L; ALT peak at H 72: 2496 U/L	α-amanitin in urine at D 4: 2.7 µgL; β-amanitin in urine on D 4: 1.25 µg/L	positive development	*Amanita phalloides*
[115]	October 18 2013	Turkey	1	M/39	NC/H 12	Nausea, vomiting, diarrhea, abdominal pain, dehydration, jaundice	Gastric lavage, activated charcoal, rehydration, *N*-acetylcysteine, antihistamine, vitamins, corticosteroid	Consumption of 5 mushrooms ≈ 19.93 mg amatoxins; ALT peak at H 90: 5124 U/L	-	positive development	*Lepiota brunneoincarnata*
[73]	1999–2015	Slovenia	32	NC	NC	NC	29 silibinin, rehydration	8 PSS1; 8 PSS2; 3 PSS3; Serum creatinine PSS3 group: 185.6 ± 40.7 µmol/L	-	1 death; 1 liver transplantation; 30 positive development	*Amanita phalloides*
[116]	April 2013	Hong Kong	7	M/48	H 12/NC	Vomiting, diarrhea	*N*-acetylcysteine, silibinin, penicillin G, activated charcoal	Serum creatinine at H 30: 229 µmol/L; ALT peak at H 48: 4856 U/L	Amatoxins identification positive in urine	positive development	*Amanita farinosa*
				F/47	H 12/NC	Vomiting, diarrhea, fever	*N*-acetylcysteine, silibinin, penicillin G, vitamin K, activated charcoal	ALT peak at H 72: 5132 U/L	Amatoxins identification positive in urine	Liver transplantation at D 5	
	March 2015			M/29	H 12/D 4	Vomiting, diarrhea, jaundice, confusion, hepatic encephalopathy	*N*-acetylcysteine, penicillin G, vitamin K, silibinin	Serum creatinine at D 4: 241 µmol/L; ALT peak at D 4: 9390 U/L	Amatoxins identification negative in urine	Liver transplantation at D 6	NC
	NC	South Africa		F/43	H 12/D 5	Vomiting, diarrhea, jaundice, confusion, tachycardia, hypotension, metabolic acidosis	Supportive care	-	-	Death at D 6	
				M/44	H 12/	Vomiting, diarrhea	*N*-acetylcysteine, activated charcoal	-	Amatoxins identification negative in urine	positive development	
		Hong Kong		M/74	H 9/D 1	Vomiting, diarrhea	*N*-acetylcysteine, silibinin, penicillin G, activated charcoal	-	Amatoxins identification positive in urine	positive development	
		China		F/40	H 8/D 4	Vomiting, diarrhea, dehydration	*N*-acetylcysteine, silibinin, penicillin G, activated charcoal	-	-	positive development	
[117]	July 2007 to August 2016	Czech Republic	23	12 M–11 F/7–78	H 2–48/H 8–60	Nausea, vomiting, diarrhea, abdominal pain, 5 hepatic encephalopathy grade I and II, 3 hepatic encephalopathy grade III and IV	Activated charcoal, rehydration, *N*-acetylcysteine, silibinin, hemoperfusion, plasmapheresis	AST: 0.5–95 U/L	-	2 deaths (1 at Mo 2 after liver transplantation); 5 liver transplantation; 16 positive development	*Amanita phalloides*
[118]	28 November 2013	China	13	13 M/19–56	H 9–21/NC	Nausea, vomiting, diarrhea, abdominal pain, fatigue, weakness, anorexia, palpitation, chest tightness, eye pain, blurred vision, leg cramps, oliguria, tachycardia	Rehydration, antiemetics, silibinin, Shenshuaining, hemodialysis	Consumption of ≈ 10–120 g of mushrooms; AST peak: 2600 U/L; ALT peak: 3581 U/L	-	positive development	*Galerina sulciceps*

N: number of patients; NC: not communicated; F: female; M: male; H: hour; D: day; Mo: month; AST: aspartate aminotransferase; ALT: alanine aminotransferase.

**Table 4:** Analytical methods for amatoxins detection.

Ref.	Matrix	Separation	Detection	Qualitative/Quantitative	LOD	LOQ	Linearity	Extraction Recovery	Additional Analytical Information
[119]	Rabbit serum	-	RIA	Qualitative	α-: 50 pg	NA	NA	NA	-
[70]	Pure substances	TLC	-	Qualitative	α-: 50 µg	NA	NA	NA	-
[120]	Mushrooms	HPTLC	Spectrophotometry	Quantitative	50 ng deposit	NC	NC	NC	-
[121]	Serum, urine, duodenal fluid, gastric juice, mushrooms	-	RIA	Quantitative	3 µg/L	NC	3.3–100 µg/L	NC	-
[122]	Serum, urine, stomach washings	HPLC	UV (280 nm)	Quantitative	10 µg/L	NC	20–500 µg/L	110%	Column: (250 mm × 4.6 mm) 5 µm Ultrasphere ODS C18; Flow rate: 1 mL/min; Mobile phase: 0.02 M ammonium acetate/ACN (88/12; *v*/*v*) pH 5; RT α-: 12.1 min, β-: 7.4 min
[123]	Serum, urine, mushrooms	HPLC	UV (302 nm)	Quantitative	10 ng	NC	0.5–20 mg/L	α-: 81.1–98.1% β-: 80.6–97.3%	Column: (125 mm × 4.0 mm) 5 µm Lichrosorb RP-18; Flow rate: 1 mL/min; Mobile phase: ACN (A), 0.01 M acetic acid-ammonium acetate buffer pH 5 (B); RT α-: 14.9 min, β-: 9.1 min
[124]	Plasma, urine	-	RIA	Quantitative	0.1 µg/L plasma 1 µg/L urines	NC	0.1–20 µg/L plasma; 1–100 µg/L urines	101.3% plasma 110% urine	-
[125]	Serum, urine	HPLC	Amperometry (Reference electrode: Ag/AgCl; Working potential: 600 mV)	Quantitative in serum; Qualitative in urine	α-: 40 pg on column β-: 80 pg on column	NC	1–1000 µg/L	α-: 53–65% β-: 36%	Column: (250 mm × 4.6 mm) 5 µm Spherisorb ODS2 - (250 mm × 4.6 mm) 5 µm Hypersil WP300 Butyl; Flow rate: 1 mL/min; Mobile phase: 0.02 M ammonium acetate/ACN (92:8; *v*/*v*) 0.5 mM EDTA pH 5; RT α-: 16.5 min, β-: 12.0 min
[126]	Plasma	HPLC	UV (303 nm)	Quantitative for α-amanitin	9.74 µg/L	10 µg/L	10–100 µg/L	67.3–105.56%	-
[127]	Plasma	HPLC	Amperometry/EC (Reference electrode: Ag/AgCl; Working potential: 350 mV)	Quantitative for α-amanitin	2 µg/L	NC	3–200 µg/L	80–82.5%	Column: (150 mm × 4.6 mm) 5 µm PLRP-S 100 Å; Flow rate: 0.5 mL/min; Mobile phase: 0.05 M phosphate buffer—ACN (91/9; *v*/*v*) pH 9.5
[128,129]	Mushrooms	HPLC	UV (214, 295 nm)	Quantitative	10 µg/L = 0.5 ng/g mushrooms	NC	NC	NC	Column: (250 mm × 4.6 mm) 5 µm Ultrasphere ODS; Flow rate: 1 mL/min; Mobile phase: 0.02 M aqueous ammonium acetate/ACN (90/10; *v*/*v* A) (76/24; *v*/*v* B)
[63]	Urine, mushrooms	Electrophoresis	DAD: 190–350 nm	Quantitative	1000 µg/L	NC	1–1000 mg/L	NC	Capillary length: 36 cm (50 µm); T separation: 25°C; Buffer: 100 mM phosphate (pH 2.4)
[130]	Urine	HPLC	Coulometry (Full scale range 50 µA until 12.5 min, 20 µA up to 20 min)	Quantitative for α-amanitin	2 µg/L	10 µg/L	10–200 µg/L	77–80.4%	Column: (250 mm × 4.6 mm) Supelcosil LC 18; Flow rate: 1 mL/min; Mobile phase: 0.005 M bisodic phosphate aqueous solution pH 7.2 and ACN (90/10; *v*/*v*); Electrode: graphite
[131]	Plasma, urine	HPLC	ESI-UV-MS (UV: 302 nm) (SIM mode (+): α- 919, 920, 921 *m*/*z*; β- 920, 921, 922 *m*/*z*)	Quantitative	2.5 µg/L	5.0 µg/L	5–75 µg/L	α-: 49.1–62.5% β-: 52.1–57.5%	Column: (100 mm × 2.1 mm) 3 µm HP ODS Hypersil RP-18; Flow rate: gradient; Mobile phase: MeOH-0.01 M ammonium acetate pH 5 (10/90; *v*/*v* A) (70/30 *v*/*v* B)
[132]	Serum, urine	ELISA	-	Quantitative for β-amanitin	0.08 µg/L	NC	0.080–2 µg/L	NC	-
[133]	Mushrooms	HPLC	HILIC-ESI-MS/MS (ion trap) (scan range: 600–930 *m*/*z*)	Quantitative	20 ng/g	α-: 26.8 ng/g β-: 33.3 ng/g	20–500 µg/L	63–75%	Column: (250 mm × 2.0 mm) 5 µm 80 Å TSK-Gel Amide 80; Flow rate: 0.2 mL/min; Mobile phase: 2 mM ammonium formate + 5mM HCOOH (A), ACN (B), MeOH (C); RT: α- ≈ 7.18 min, β- ≈ 8.94 min
[134]	Serum, liver	HPLC	ESI-MS/MS/MS (ion trap) (α- 941 to 746 (CE 40%) *m*/*z*; Full-scan of product ions of *m*/*z* 746 (CE 25%))	Quantitative for α-amanitin	0.26 ng/g (serum) 0.5 ng/g (liver)	NC	1–50 µg/L	95% (serum) 98% (liver)	Column: (100 mm × 4.6 mm) Synergi RP-Polar; Flow rate: 0.5 mL/min; Mobile phase: 0.01 M ammonium acetate in H_2_O 0.1% HCOOH (A), 0.01 M ammonium acetate in MeOH 0.1% HCOOH (B); RT: α-: 4.5 min
[135]	Urine	Electrophoresis	DAD (214 nm)	Quantitative	2.5 µg/L	5 µg/L	5 - 100 µg/L	NC	Capillary length: 48 cm (75 µm); T separation: 25 °C
[136]	Plasma	HPLC	ESI-MS/MS (ion trap) (SIM mode: α- 919–921 *m*/*z*; β- 920–922 *m*/*z*)	Quantitative	0.5 µg/L	NC	10–500 µg/L	77–79%	Column: (150 mm × 2.0 mm) Capcell Pak C18 UG120; Flow rate: 0.2 mL/min; Mobile phase: H_2_O 0.1% HCOOH (A), ACN 0.1% HCOOH (B); RT: α-: 19.0 min, β-: 20.1 min
[137]	Mushrooms	HPLC	ESI-TOF-MS (Full-scan: 100–1000 *m*/*z*)	Quantitative	30 ng/g	NC	100–1000 ng/g	53.1–69.6%	Column: (150 mm × 2.0 mm) 3 µm TSK-gel Amide-80; Flow rate: 1 mL/min; Mobile phase: ACN (A), 15% MeOH in 10 mM ammonium acetate (B)
[11]	Serum, urine	UPLC	ESI-MS/MS (triple Q) (α- 919.6 to 919.6 (20 eV) *m*/*z*; β-: 920.6 to 920.6 (20 eV) *m*/*z*)	Quantitative	0.5–1.5 µg/L	NC	2–420 µg/L	91.3–110%	Column: (100 mm × 2.1 mm) 1.7 µm ACQUITY BEH Shield RP18; Flow rate: 0.4 mL/min; Mobile phase: H_2_O 0.1% HCOOH (A), MeOH (B); RT: α-: 2.23 min, β-: 2.49 min
[138]	Urine	MALDI	ESI-TOF-MS-MS	Quantitative	0.5 µg/L	NC	10–500 µg/L	60–80%	-
[139]	Urine, liver	UPLC	ESI-MS/MS (triple Q) (α-: 919.48 to 259.13 (44 eV)/919.48 to 901.53 (28 eV) *m*/*z*; β-: 920.48 to 259.13 (42 eV)/920.48 to 902.44 (26 eV) *m*/*z*)	Quantitative	0.20 µg/L (urine) 10 ng/g (liver)	0.46–0.57 µg/L (urine) 12.3–14.7 ng/g (liver)	10–200 µg/L (et ng/g)	90.4–105.0% (urine) 90.2– 12.9% (liver)	Column: (100 mm × 2.1 mm) 1.8 µm ACQUITY HSS T3; Flow rate: 0.5 mL/min; Mobile phase: 0.02 M ammonium acetate pH 5 (A), ACN (B); RT: α-: 5.73 min, β-: 5.27 min
[140]	Urine	UPLC	(-) ESI-HR/MS/MS (orbitrap) (SIM mode: α-: 917.3458 *m*/*z*; β-: 918.3298 *m*/*z*)	Quantitative for α-amanitin	1 µg/L	1 µg/L	1–100 µg/L	64–102%	Column: (150 mm × 2.1 mm) 2.6 µm TF Accucore PhenylHexyl; Mobile phase: 10 mM ammonium acetate in H_2_O 0.01% HCOOH pH 5 (A), ACN 0.1% HCOOH (B), 2-propanol/ACN (1:1; *v*/*v*) (C); RT: α-: 8.23 min, β-: 7.61 min
[141]	Urine	UPLC	HR/MS/MS (orbitrap) (SIM mode: α-: 919.3614 *m*/*z*; β-: 920.3455 *m*/*z*)	Quantitative	α-: 0.25 µg/L β-: 0.5 µg/L	α-: 0.5 µg/L β-: 0.75 µg/L	1–100 µg/L	88.4–93.4%	Column: (100 mm × 2.1 mm) 2.6 µm Accucore C18; Flow rate: 0.4 mL/min; Mobile phase: 10 mM ammonium acetate buffer 0.1% HCOOH (A), ACN 0.1% HCOOH (B); RT: α-: 1.9 min, β-: 1.7 min
[142]	Mushrooms	HPLC	DAD (303 nm)	Quantitative	2 ng/g	NC	NC	NC	Column: (150 mm × 4.6 mm) 5 µm C18; Flow rate: 1 mL/min; Mobile phase: 0.05 M ammonium acetate pH 5.5 with HCOOH/ACN (90:10; *v*/*v*)
[143]	Urine	UPLC	ESI-TOF/MS (Full-scan 50–1000 *m*/*z*)	Quantitative	1 µg/L	NC	1–1000 µg/L	86–98%	Column: (100 mm × 2.1 mm) 2.2 µm Acclaim RS 120, C18; Flow rate: 0.2 mL/min; Mobile phase: H_2_O/ACN (99/1; *v*/*v*) 2mM ammonium formate, 0.1% HCOOH (A), ACN/H_2_O (99/1; *v*/*v*) 2mM ammonium formate, 0.1% HCOOH (B); RT: α-: 6.05 min, β-: 6.08 min
[144]	Rat liver and kidney Serum	HPLC	DAD-EC (UV: 305 nm)	Quantitative for α-amanitin	UV: 110 ng/g (liver) 160 ng/g (kidney) EC: 70 ng/g (liver) 40 ng/g (kidney)	UV: 330 ng/g (liver) 500 ng/g (kidney) EC: 210 ng/g (liver) 110 ng/g (kidney)	UV: 330–10000 µg/L (liver) 500–10000 µg/L (kidney) EC: 210–10000 µg/L (liver) 110–10000 µg/L (kidney)	UV: 99.4% (liver) 100% (kidney) EC: 98.8% (liver) 99.7% (kidney)	Column: (250 mm × 4.6 mm) 5 µm Spherisorb RP-18 ODS2; Flow rate: 1 mL/min; Mobile phase: 20% MeOH in 50 mM citric acid, 0.46 mM octanessulfonic acid pH 5.5 with 10 M NaOH
[145]	Serum, urine	UPLC	ESI-MS/MS (triple Q) (α-: 919.5 to 259.1 (42 eV)/919.5 to 86.0 (68 eV) *m*/*z*; β-: 920.5 to 259.1 (42 eV)/920.5 to 86.0 (71 eV) *m*/*z*)	Quantitative	0.5–1 ng/g	1–2.5 ng/g	1–100 µg/L	80.7–88.6%	Column: (100 mm × 2.1) 1.6 µm; Flow rate: 0.2 mL/min; Mobile phase: 0.2% HCOOH in H_2_O (A), 0.2% HCOOH in MeOH (B); RT α-: 4.72 min, β-: 4.96 min
[146]	Food with mushrooms	HPLC	(-) ESI-MS/MS (triple Q) (α-: 917.4 to 205.1/917.4 to 257.1 *m*/*z*; β-: 918.4 to 205.1/918.4 to 257.1 *m*/*z*)	Quantitative	5 ng/g	10 ng/g	10–2000 ng/g	77.6–90.4%	Column: (150 mm × 3.0 mm) 2.5 µm XBridge™ BEH C18; Flow rate: 0.3 mL/min; Mobile phase: MeOH (A), 0.03% ammonia solution in H_2_O pH 10.5 (B)
[147]	Rat plasma	HPLC	(+) ESI-MS/MS (triple Q) (MRM: 919.45 to 259.20 (47 eV); 919.45 to 901.45 (26 eV); 919.45 to 86.15 (50 eV) *m*/*z*)	Quantitative for α-amanitin	3.0 µg/L	8.5 µg/L	10–1500 µg/L	85–115%	Column: (100 mm × 2.1 mm) 5 µm Hypersil GOLD C18; Flow rate: 0.2 mL/min; Mobile phase: 0.02 mol/L ammonium acetate, 0.1% HCOOH (A), ACN (B); RT: 4.86 min
[148]	Rat plasma and urine	HPLC	PDA-MS/MS/MS (IT-TOF) (PDA scan: 190–400 nm; Full-scan: 700–1000 *m*/*z*; Multiple stage fragmentation: 100–900 *m*/*z* for MS^2^, 50–900 *m*/*z* for MS^3^)	Qualitative	NC	NA	NA	NA	Column: (100 mm × 2.1 mm) 3µm Inertsil ODS-3; Flow rate: 0.2 mL/min; Mobile Phase: 20 mM ammonium acetate, 0.1% HCOOH (A), ACN (B); RT α-: 11.05 min, β-: 10.20 min
[149]	Urine	HPLC	ESI-MS/MS (triple Q) (α-: 919.3 to 338.9 *m*/*z*; ^15^N_10_- α-: 929.3 to 911.4 *m*/*z*, β-: 920.3 to 644.3 *m*/*z*)	Quantitative with ^15^N_10_-α-amanitin	α-: 0.458 µg/L β-: 0.930 µg/L	NC	α-:1–200 µg/L β-: 2.5–200 µg/L	α-: 97.8% β-: 71.1%	Column: (50 mm × 2.1 mm) 1.7 µm Acquity BEH HILIC; Flow rate: gradient; Mobile phase: 10 mM ammonium formate in ACN (25/75; *v*/*v*) 1% HCOOH (A), 10 mM ammonium formate in ACN (10/90; *v*/*v*) 0.2% HCOOH (B)
[56]	Standard solution	-	PSI-HR-MS/MS (α-: 919.3610 to 86.0606 *m*/*z*; β-: 920.3405 to 86.0606 *m*/*z*)	Qualitative	NA	NA	NA	NA	-
[150]	Mushrooms	-	LFIA	Qualitative	α-: 10 µg/L β-: 2000 µg/L γ-: 10 µg/L	NA	NA	NA	-

NA: not applicable; LOD: limit of detection; LOQ: limit of quantification; NC: not communicated; RT: retention time; DAD: diode array detection; EC: electrochemical.

**Table 5:** Cases of ibotenic acid, muscimol, and muscarine poisoning.

Ref.	Date of Intoxication	Country	*N*	Sex/Age	Offset of Symptoms/Delay before Hospitalization	Symptoms	Treatment	Notes	Toxin Quantification	Outcome	Mushroom Specie
[162]	NC	South Africa	4	M/62	H 0.5/H1.5	Dizziness, tiredness, clouding vision, vomiting, disorders of the state of consciousness, miosis, salivation, twitching, agitation, visual hallucinations	Atropine, diuresis, gastric lavage, rehydration, antibiotic, sedative, analgesic	Consumption of 2 tablespoonful	-	Positive development with mental deficit for 6 weeks	*Amanita pantherina*
				F/51		Dizziness, tiredness, nausea, miosis	Gastric lavage, atropine, rehydration, antibiotic, sedative, analgesic				
				M/16		Dizziness, tiredness, clouding vision, nausea, vomiting, salivation, twitching	Gastric lavage, atropine, rehydration, sedative, analgesic				
				M/23	H 1/NC	Twitching, tiredness, visual problem, disorders of the state of consciousness, salivation, severe respiratory embarrassment	Gastric lavage, atropine, rehydration, analeptics, antibiotic, tracheostomy, sedative, analgesic				
[163]	20 July 1964	United States, Massachusetts	1	M/58	H 2/H 4	Nausea, vomiting, diarrhea, salivation, blurred vision, twitching, disorientation, disorders of the state of consciousness	Gastric lavage, glucose, atropine	Obesity, concomitant consumption of alcohol	-	Positive development	*Amanita crenulata*
[164]	NC	Finland	3	F/27	H 2/NC	Nausea, vomiting, vertigo, twitching, hallucinations, loss of consciousness, salivation, hypothermia	Gastric lavage, activated charcoal, glucose	Confusion with *Macrolepiota* procera; Consumption of fried mushrooms	-	Positive development	*Amanita regalis*
				M/55	H 2/H 4	Nausea, vomiting, disorientation, hallucinations, sudation, hypothermia	Activated charcoal	Past of inferior myocardial infarction, renal insufficiency, glaucoma; Consumption of about 2 cooked mushrooms, confusion with *Macrolepiota procera*			
				F/53	H 1/H 3	Vomiting	Activated charcoal	Confusion with *Macrolepiota procera,* consumption of cooked mushrooms			
[165]	17 December 1980	Zimbabwe	2	M/10	NC/NC	Nausea, vomiting, dizziness, disorders of the state of consciousness, twitching, mydriasis	Glucose	Consumption of a handful of mushrooms	-	Positive development	*Amanita pantherina*
				F/20	H 0.33/NC	Nausea, epigastric discomfort, blurred vision, drowsiness, confusion, twitching	Dextrose, diuretic, atropine	Consumption of cooked mushrooms			
[166]	27 September 1981	United states, New York	1	M/58	H 1.5/H 2.25	Nausea, vomiting, diarrhea, sudation, confusion, agitation, disorientation, visual hallucinations	Rehydration, gastric lavage, activated charcoal	Consumption of cooked mushrooms	-	Positive development	*Amanita muscaria*
[167]	NC	United States, Missouri	5	4 M, 1F/NC	H 1/NC	Vomiting, diarrhea, abdominal cramps, salivation, diaphoresis, tiredness, weakness, mydriasis, blurred vision, bradycardia	Atropine	-	-	Positive development	*Amanita muscaria* suspected
[168]	1979–1989; Between 6 April 6 and 23 May	United States, Washington	11	8 M, 3 F/11 months to 20 YO	NC	Vomiting, incoherent babbling, confusion, irritability, hysteria, hallucinations, myoclonic jerking, lethargy, ataxia, bradycardia, mydriasis	Syrup of Ipecac, gastric lavage, charcoal, anticonvulsants, atropine	1 voluntary consumption seeking hallucinogenic experience; 1 autistic male	-	Positive development	*Amanita pantherina, Amanita muscaria*
[169]	NC	Poland	5	F/18	H 0.33/H 5	Auditory and visual hallucinations, tiredness, gastric pain, loss of consciousness	Activated charcoal, antidiarrheal, potassium chloride	Voluntary consumption seeking hallucinogenic experience, concomitant consumption of alcohol	-	Positive development	*Amanita muscaria*
[170]	NC	Australia	1	F/53	H1/H3	Headache, chest and abdominal pain, vomiting, diarrhea, sweating, confusion, hypotension, bradycardia, metabolic and respiratory acidosis	Intubation, rehydration, atropine, adrenaline, noradrenaline, metaraminol, glucagon, activated charcoal, dialysis	Consumption of 2 mushrooms		Death at H10	*Rubinoboletus* sensu lato pro tempe
[171]	NC	Poland	2	F/47	H2/NC	Nausea, abdominal pain, vomiting, diarrhea, agitation, vertigo, paresthesia of left arm, mystical experiences, speech disorder	NC	Confusion with *Macrolepiota* procera; Consumption of 5 mushrooms	-	Positive development	*Amanita pantherina*
				F/27	H2/H3	Nausea, abdominal pain, vomiting, diarrhea, dizziness, anxiety, humming in head	Activated charcoal, laxatives, infusions, electrolytes supplementation				
[9]	NC	Slovenia	1	M/48	H1.5/H4	Nausea, vomiting, somnolence, disturbance of consciousness, myoclonus, hypothermia, tachycardia, confusion, visual and auditory hallucinations and paranoia at H18	Activated charcoal, midazolam, olanzapine	Confusion with *Amanita caesarea*	-	paranoid psychosis with auditory and visual hallucinations for 5 days	*Amanita muscaria*
[172]	05 October 2005	France	2	M/67	H 2/H 15	Vomiting, abdominal pain, diarrhea, sudation, miosis	Rehydration, activated charcoal, laxative, atropine	Medical history of arterial hypertension, dyslipidemia, renal colic	-	Positive development	*Inocybe patouillardii*
				F/67	H 2/H 15	Vomiting, abdominal pain, diarrhea, sudation, miosis, disturbance of consciousness, cardiac arrest, hypothermia, tachycardia	Intubation, adrenaline, atropine, antibiotic, anticonvulsant	Medical history of diabetes, arterial hypertension, dyslipidemia, hypothyroidism, restrictive respiratory failure secondary to obesity	-	Death of postanoxic encephalopathy at J 7	
[173]	November 2006 to January 2008	Israel	14	8–60	H 0.25–2/NC	Nausea, vomiting, abdominal pain, diarrhea, diaphoresis, salivation, lacrimation, tachycardia, blurred vision, miosis	Rehydration, antiemetic, atropine	Confusion with *Suillus granulatus* and *Tricholoma terreum*; Consumption of cooked mushrooms	-	Positive development	*Inocybe fastigiata, I. geophylla, I. patouillardii*
[174]	Autumn 2006	Turkey	1	M/11	H 2/NC	Vomiting, abdominal pain, diarrhea, salivation	NC	Confusion with *Russula* sp.; Consumption of cooked mushrooms	-	Death at D 4	*Inocybe rimosa*
[175]	2010	France	23	M/59	H 1/NC	Nausea, vomiting, abdominal pain, sweating, motor and sensory deficit in the lower limbs, bradycardia, miosis, hypothermia, dehydration, functional renal failure, occlusive thrombosis	Atropine, surgery for the occlusive thrombosis	Medical history of bi-femoral bypass surgery in 1989	-	Positive development	NC
				F/76	H 0.5/NC	Vomiting, diarrhea, sweating, bradycardia, cardiovascular collapse, miosis, hypothermia, dehydration, functional renal failure	Atropine	Medical history of lower limb arteriopathy obliterans			
[155]	NC	Czech Republic	1	M/55	NC/NC	NC	NC	-	In urine: muscarine: 0.045 mg/L	Death	*Amanita muscaria*
[176]	NC	Czech Republic	4	F/28	H 1.5/NC	Vomiting, hallucinations	Gastric lavage, activated charcoal, intubation	-	In urine: IBO at H 4: 47.7 mg/L; MUS at H 4: 9.9 mg/L	Positive development	*Amanita pantherina*
				M/66	NC/NC	dizziness	Gastric lavage, activated charcoal	Confusion with *Amanita rubescens*	In urine: IBO at H 8: 32.2 mg/L; MUS at H 4: 6.0 mg/L		
				M/62	NC/H 6	Diarrhea, agitation, incoherence	NC	-	In urine: IBO at H 6: 55.2 mg/L; MUS at H 6: 7.4 mg/L		
				F/62	NC/H 2.5	Nausea, vomiting, hallucinations	Activated charcoal, laxative, diuresis	-	In urine: IBO at H 3: 37.3 mg/L; MUS at H 3: 7.6 mg/L		
[177]	NC	Japan	1	M/59	NC/NC	NC	NC	-	In serum: IBO: 95.9 µg/L; MUS: 105 µg/L	Positive development	*Amanita ibotengutake*
[178]	Springtime	Poland	1	M/21	NC/NC	Unconscious, seizure, mydriasis, salivation, hyperthermia	Intubation, gastric lavage, rehydration	Voluntary consumption seeking hallucinogenic experience; Stop his treatment for depression; Consumption of marijuana	-	Positive development	*Amanita muscaria*

N: number of patients; NC: Not communicated; F: female; M: male; H: hour; D: day; IBO: ibotenic acid; MUS: muscimol.

**Table 6:** Analytical methods for muscarine detection.

Ref.	Matrix	Separation	Detection	Qualitative/Quantitative	LOD	LOQ	Linearity	Extraction Recovery	Additional Analytical Information
[179]	Mushrooms	TLC	Reactant of Thies and Reuther	Quantitative	6 µg	NC	NC	NC	-
[180]	Mushrooms	TLC	SIMS-MS	Qualitative	10 µg deposit	NA	NA	NA	-
		HPLC	UV (254 nm)	Qualitative	NC	NA	NA	NA	Column: (250 mm × 4.6 mm) 10 µm Lichrosorb RP-8; Mobile phase: H_2_O 1% glacial acetic acid (A), ACN (B)
		HPLC	MS/MS (triple Q)	Qualitative	NC	NA	NA	NA	-
[133]	Mushrooms	UPLC-HILIC	ESI-MS/MS (ion trap) (Scan range: 90–180 *m*/*z*)	Quantitative	5 ng/g	5.1 ng/g	5–50 µg/L	84–94%	Column: (250 mm × 2.0 mm) 5 µm 80 Å TSK-Gel Amide 80; Flow rate: 0.2 mL/min; Mobile phase: 2 mM ammonium formate + 5 mM HCOOH (A), ACN (B), MeOH (C); RT: ≈ 9.5 min
[158]	Urine	HPLC	ESI-MS (Full-scan mode)	Qualitative	3 µg/L	NC	NC	90%	Column: (150 mm × 2.0) 5 µm Gemini C18; Flow rate: 0.2 mL/min; Mobile phase: 8 mmol/L heptafluorobutyric acid in H_2_O; RT: 14.2 min
[155]	Urine	HPLC	ESI-MS	Quantitative	0.09 µg/L	0.3 µg/L	0.3–2000 µg/L	95–96%	Column: (150 mm × 2.0 mm) 5 µm Gemini C18; Flow rate: 0.2 mL/min; Mobile phase: 8 mmol/L heptafluorobutyric acid in H_2_O (A), ACN (B); RT: 10.0 min
[181]	Mushrooms	HPLC	ESI-MS/MS (triple Q) (SRM mode: 174 to 57;174 to 115; 174 to 60;174 to 97 *m*/*z*)	Quantitative	NC	NC	NC	NC	Column: (150 mm × 2.0 mm) 5 µm 110 Å Gemini C18; Flow rate: 0.15 mL/min; Mobile phase: H_2_O (A), ACN (B); RT: 1.8 min
[182]	Urine	Electrophoresis	ESI-MS/MS (triple Q) (SIM and MRM mode)	Quantitative	0.73 µg/L	NC	0.1–10.00 mg/L	92.6–95.4%	Capillary length: 100 cm (50 µm); Sheath liquid: H_2_O/MeOH/CH_3_COOH (20/79.65/0.35 *v*/*v*/v/); Flow rate: 0.4 mL/min
[143]	Urine	UPLC	ESI-TOF/MS (Full-scan 50–1000 *m*/*z*)	Quantitative	0.09 µg/L	NC	0.1–100 µg/L	97%	Column: (100 mm × 2.1 mm) 2.2 µm Acclaim RS 120, C18; Flow rate: 0.2 mL/min; Mobile phase: H_2_O/ACN (99/1; *v*/*v*) 2 mM ammonium formate, 0.1% HCOOH (A), ACN/H_2_O (99/1; *v*/*v*) 2 mM ammonium formate, 0.1% HCOOH (B); RT: 2.05 min
[56]	Standard solution	-	PSI-HR-MS/MS (α-: 174.1486 to 174.1486 *m*/*z*)	Qualitative	NA	NA	NA	NA	-

NA: not applicable; LOD: limit of detection; LOQ: limit of quantification; NC: not communicated; RT: retention time.

**Table 7:** Analytical methods for ibotenic acid and muscimol detection.

Ref.	Matrix	Separation	Detection	Qualitative/Quantitative	LOD	LOQ	Linearity	Extraction Recovery	Additional Analytical Information
[199]	Mushrooms	GC	MS	Quantitative	NC	NC	NC	NC	Columns: (0.75 m × 2.8 mm) OV-101 and (1.2 m × 2.8 mm) SE-30; Helium flow rate: 20 mL/min; T transfer line: 175 °C
[200]	Mushrooms	HPLC	UV (440, 570 nm)	Quantitative	30 ng	NC	NC	NC	Column: (350 mm × 2.7 mm); RT IBO: 11 min, MUS: 83 min
[188]	Mushrooms	HPLC	UV (210 nm)	Quantitative	1 ppm	NC	NC	<98%	Column: (25 mm × 4.0 mm) IRICA RP-18T; Flow rate: 0.6 mL/min; Mobile phase: H_2_O/ACN/MeOH (65:20:15; *v*/*v*/v) with 2.1 mM sodium dodecyl sulfate + 4 mM H_3_PO_4,_ isocratic mode
[201]	Mushrooms	HPLC	UV (230, 254 nm)	Quantitative	18 µg/L IBO 0 µg/L MUS	NC	50–1000 µg/L IBO 100–3000 µg/L MUS	NC	Column: (250 mm × 4.6 mm) 5 µm Spherisorb S5 ODS-2; Flow rate: 0.1 mL/min; Mobile phase: 5 mM octylammonium o-phosphate
[202]	Mushrooms	HPLC	PDA	Quantitative just of IBO	NC	NC	NC	NC	Preparative column IBO: (115 mm × 13 mm) C18; Flow rate IBO: 0.5 mL/min; RT IBO: 8.2 min; Column MUS: (150 mm × 4.6) Zorbax SB-Aq; Flow rate MUS: 1.0 mL/min; RT MUS: 12.8 min; Mobile phase: H_2_O/ACN/MeOH (65:20:15; *v*/*v*/v) with 2.1 mM sodium dodecyl sulfate + 4 mM H_3_PO_4_, isocratic mode
		HPLC	UV-MS (UV: 254 nm)						Column: (100 mm × 2.1 mm) 5 µm XTerra^TM^ MS C18; Flow rate: 0.5 mL/min; Mobile phase: H_2_O/MeOH (19:1; *v*/*v*) to ACN/H_2_O/MeOH (18:1:1; *v*/*v*/v)
[203]	Mushrooms	HPLC	ESI-MS/MS (triple Q) (IBO: 159 to 113.1;159 to 42.3 *m*/*z*; MUS: 115.1 to 98.1; 115.1 to 67.2; 115.1 to 39.4 *m*/*z*)	Quantitative	NC	NC	NC	NC	Column: (150 mm × 2.1 mm) 5 µm Uptisphère ODB C18; Flow rate: 0.2 mL/min; Mobile phase: 2mM ammonium formiate buffer pH 3 (A), ACN (B)
[189]	Mushrooms	GC	MS (SIM: IBO: 257 *m*/*z*, MUS: 243 *m*/*z*)	Quantitative IBO/MUS	NC	NC	10–400 ppm IBO 25–2000 ppm MUS	NC	Column: (30 m × 0.25 mm) 0.25 µm DB-5 ms; Helium flow rate: 53 mL/min; T injector: 250 °C; Toven: 100 °C
[204]	Mushrooms	HPLC	UV (256 nm)	Quantitative	7.8 ppm IBO 1.4 ppm MUS	25.9 ppm IBO 4.6 ppm MUS	40–2500 ppm IBO 25–2500 ppm MUS	95.4–101.1%	Column: (150 mm × 2.1 mm) 3.5 µm Symmetry C18; Flow rate: 0.2 mL/min; Mobile phase: 10 mM ammonium acetate (A), ACN (B); RT IBO: 25.92 min, MUS: 24.65 min
		LC	ESI-MS/MS (ion trap) (IBO: 419 to 355; 419 to 235; 419 to 183 *m*/*z*; MUS: 347 to 317; 347 to 276; 347 to 226; 347 to 183 *m*/*z*)	Qualitative	25 ppm	NA	NA	NA	
[158]	Urine	HPLC	ESI-MS (Full-scan mode)	Qualitative	50 µg/L IBO 40 µg/L MUS	NC	NC	15% IBO 22% MUS	Column: (150 mm × 2.0 mm) 5 µm Gemini C18; Flow rate: 0.2 mL/min; Mobile phase: 8 mmol/L heptafluorobutyric acid in H_2_O; RT: IBO 2.6 min, MUS 4.6 min
[176]	Urine	GC	MS (Full Scan: 40–400 *m*/*z* and SIM: MUS: 113 *m*/*z*; IBO: 257 *m*/*z*)	Quantitative	1 mg/L	NC	1–15 mg/L	74% IBO 80% MUS	Column: (15 m × 0.25 mm) 0.25 µm HP-5MS; Helium flow rate: 1.5 mL/min; T injector: 220 °C; T transfer line: 250 °C
[186]	Mushrooms	LC-HILIC	ESI-MS/MS (triple Q) (IBO: 159 to 113.1 *m*/*z*; MUS: 115 to 98.1 *m*/*z*)	Quantitative	<10 µg/g	NC	10–500 µg/g	84.6–107%	Column: (150 mm × 2.0 mm) 3 µm TSK-GEL Amide-80; Flow rate: 0.5 mL/min; Mobile phase: H_2_O 0.5% HCOOH (A), ACN 0.5% HCOOH (B)
[177]	Serum	LC-HILIC	ESI-MS/MS (triple Q) (IBO: 159 to 113.1 *m*/*z*; MUS: 115 to 98.1 *m*/*z*)	Quantitative	1 µg/L IBO 2.5 µg/L MUS	NC	10–1000 µg/L	87.9–103%	Column: (150 mm × 2.0 mm) 3 µm TSK-GEL Amide-80; Flow rate: 0.5 mL/min; Mobile phase: H_2_O 0.5% HCOOH (A), ACN 0.5% HCOOH (B)
[187]	Mushrooms	Electrophoresis	PDA (214 nm)	Quantitative	1.5 µg/g IBO 1.8 µg/g MUS	4.6 µg/g IBO 5.4 µg/g MUS	2.5–7000 mg/L	87–95%	Capillary length: 57 cm (75 µm); Running buffer: 25 mM sodium phosphate pH 3 (5:95; *v*/*v*)
[182]	Urine	Electrophoresis	ESI-MS/MS (triple Q) (SIM and MRM mode)	Quantitative	0.15 µg/L IBO 0.05 µg/L MUS	NC	10–1000 µg/L	92.6–95.4%	Capillary length: 100 cm (50 µm); Flow rate: 0.4 mL/min; Sheath liquid: H_2_O/MeOH/CH_3_COOH (20/79.65/0.35; *v*/*v*/v)
[205]	Urine	NMR	-	Quantitative	30 mg/L IBO 3 mg/L MUS	NC	2–417 mg/L IBO 3–278 mg/L MUS	NC	-
[56]	Standard solution	-	PSI-HR-MS/MS (IBO: 159.0397 to 113.0348 *m*/*z* MUS: 115.0504 to 98.0241 *m*/*z*)	Qualitative	NA	NA	NA	NA	-

NA: not applicable; LOD: limit of detection; LOQ: limit of quantification; NC: not communicated; RT: retention time; IBO: ibotenic acid; MUS: muscimol.

**Table 8:** Cases of gyromitrine poisoning.

Ref.	Date of intoxication	Country	*N*	Sex/Age	Offset of symptoms/Delay before hospitalization	Symptoms	Treatment	Notes	Toxin Quantification	Outcome	Mushroom specie
[214]	11 May 1935	United States, Michigan	7	F/69	NC/D 1	Vomiting, severe chest and legs pain, fever, tachycardia, convulsions, coma	Morphine, atropine, stomach wash, caffeine, sodium benzoate	Consumption of dried mushrooms after having been parboiled	-	Death at D 5	*Gyromitra esculenta*
[215]	Between 1782 and 1965	Eastern Europe	Minimum of 654	-	-	Gastrointestinal disorders	NC	-	-	At least 114 death	*Gyromitra esculenta*
[216]	9 June 1962	France	1	F/8	D 3/NC	Vomiting, agitation, delirium, bilateral mydriasis, coma, muscular hypertonia, arterial hypertension	NC	Consumption on 2 occasions	-	Death of liver failure	*Gyromitra esculenta*
	April 1964		3	F/7	H 12/NC	Vomiting, subictus, delirium, agitation, coma, oliguria, fever, respiratory collapse, liver failure	Tracheotomy, artificial ventilation	Consumption several times over 3 weeks	-	Death of liver failure at H 102	
				F/4		Vomiting, liver failure	NC			Positive development	
				F/NC		Vomiting, asthenia, subictus, liver failure				Positive development	
	Between 1817 and 1965	NC	282	NC/NC	NC/NC	Vomiting	NC	-		21 death	
[206]	NC	Italy	1	F/53	D 1/D 1	Vomiting, diarrhea, jaundice, hypotension, anuria, severe enlargement of the liver, right hemiplegia, coma	Plasma infusion, corticosteroids	Autopsy: liver necrosis, brain oedema,	TLC on intestine extract	Death at D 3	*Gyromitra esculenta*
[212]	Springtime	Canada	2	F/49	H 2/D 1	Nausea, vomiting, abdominal pain, hot and cold chills, fatigue, anorexia, jaundice	Rehydration, analgesic, antiemetic, Vitamin B6, antacid, antihistamine	AST on D 5: 431 U/L; ALT on D 5: 472 U/L	-	Positive development	*Gyromitra esculenta*
				M/56	NC/D 1	Nausea, vomiting, abdominal pain, jaundice, headache		AST on D 4: 116 U/L	-		

N: number of patients; NC: not communicated; F: female; M: male; H: hour; D: day; AST: aspartate aminotransferase; ALT: alanine aminotransferase.

**Table 9:** Analytical methods for gyromitrine detection.

Ref.	Matrix	Separation	Detection	Qualitative/Quantitative	LOD	LOQ	Linearity	Extraction Recovery	Additional Analytical Information
[206]	Viscera	TLC	UV (254–277 nm) IR (NC)	Qualitative and quantitative	NC	NC	0.1–0.5 g/L	NC	-
[219]	Mice gastric content	GC	UV and IR	Quantitative	NC	NC	NC	NC	Column: (2 mm × 2 mm) Chromosorb 103; T column: 160 °C; Helium flow rate: 20 mL/min; RT: GYRO: 17 min, MFH: 15.7 min
[220]	Mushrooms	GC	MS	Quantitative	NC	NC	NC	NC	Column: 50 m FFAP
[221]	Mice peritoneal fluids	GC	MS	Quantitative (MH)	NC	NC	NC	NC	-
[222]	Mushrooms	TLC	Spectrofluorimetry (λ_excitation_ = 340 nm; λ_emission_ = 610 nm)	Quantitative	NC	NC	0.43–2.17 ng	NC	-
[223]	Mushrooms	GC	FID	Quantitative	NC	NC	NC	30–74% GYRO 96–124% MH	Column: (25 mm × 0.31 mm) SE-54; Helium flow rate: 1 mL/min; RT: 7.3 min
[224]	Mushrooms	GC	EI-MS (Full-scan 35–650 *m*/*z*)	Quantitative	MH: 12 µg/L = 0.3 µg/g of gyromitrin	NC	NC–1.2 mg/L	36–55%	Column: (30 mm × 0.25 mm) 0.25 µm HP5-MS
[56]	Standard solution	-	PSI-HR-MS/MS (101.0713 to 73.0764 *m*/*z*)	Qualitative	NA	NA	NA	NA	-

NA: not applicable; LOD: limit of detection; LOQ: limit of quantification; NC: not communicated; GYRO: gyromitrin; MFH: *N*-methyl-*N*-formylhydrazine; MH: methylhydrazine.
